# Modeling, Simulation, and Membrane Wetting Estimation in Gas–Liquid Contacting Processes Including Shell-Side Reaction: Biogas Upgrading Using DEA Solution

**DOI:** 10.1021/acs.iecr.4c00525

**Published:** 2024-05-09

**Authors:** Grigorios Pantoleontos, Dimitrios Koutsonikolas, Akrivi G. Asimakopoulou, Souzana Lorentzou, George Karagiannakis

**Affiliations:** Advanced Renewable Technologies & Environmental Materials in Integrated Systems (ARTEMIS) Laboratory, Chemical Process & Energy Resources Institute, Centre for Research & Technology Hellas (CPERI/CERTH), 6th km Charilaou-Thermi, P.O. Box 361, Thermi, Thessaloniki 57001, Greece

## Abstract

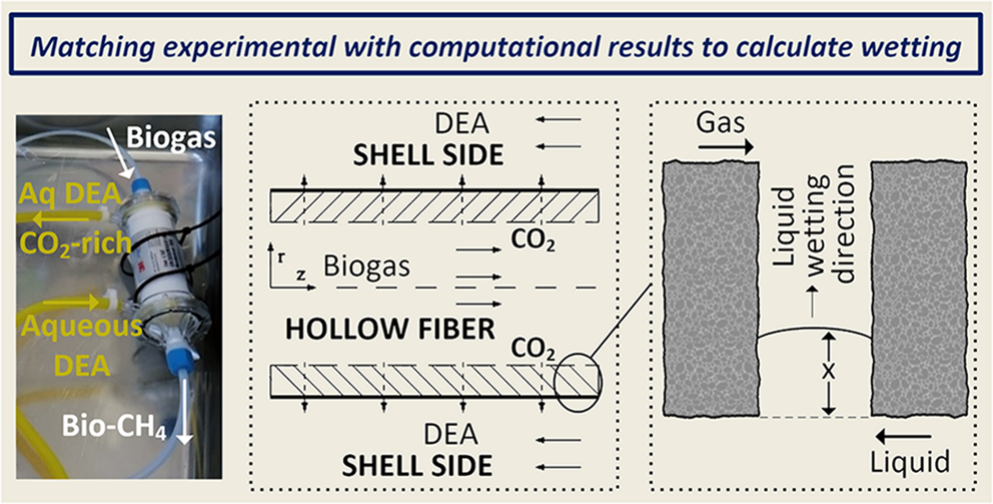

The basic principles of a steady-state mass transfer model and the resistance-in-series film model are assessed with the aid of a series of experiments in a gas–liquid contact membrane mini-module (3 M Liqui-Cel MM-1.7 × 5.5) using an aqueous solution of diethanolamine (DEA) of 0.25 M (mol/L) for biogas upgrading. Experimental data show that CO_2_ removal may exceed 67% and reach 100% in combination with the highest possible recovery of CH_4_ when employing biogas flow rates in the range of 2.8 × 10^–5^ – 3.6 × 10^–5^ m^3^/s and solvent flow rates within 0.47 × 10^–5^ – 0.58 × 10^–5^ m^3^/s. For the experimental data set, a correlation has been developed, effectively interpolating CO_2_ removal with the gas and liquid flow rates. The wetting values calculated are concentrated close to each other for the same liquid flow rate without considerably depending on the gas flow rate, especially when applying the Hikita–Yun (reaction rate–shell-side correlation) compared with the Hikita–Costello pair. Furthermore, the calculated wetting diminishes with increasing liquid flow rate, a result that is consistent with previous modeling attempts and relevant literature indications. The assumption of enhanced mass transfer in the liquid-filled part of the membrane pores due to the reaction is scrutinized, leading to objectionable computational wetting values. It is shown that for a concentration of DEA equal to 0.25 M the Hatta numbers and the enhancement factors are not equal in the whole reaction path; thus, the choice of the shell-side correlation has an appreciable impact on the overall analysis, especially for the determination of the wetting values.

## 1 Introduction

Existing CO_2_ capture technologies are primarily based on solvent absorption, such as amines in packed columns or in pressure swing adsorption (PSA) processes. Such applications have significant energy consumption and require large equipment for solvent circulation and heat transfer, resulting in reduced process efficiency and increased operating expenses. Biogas, a renewable energy source, is a gas mixture typically consisting of methane (∼50 to 60%), carbon dioxide (∼40 to 50%), and other species (e.g., H_2_S < 1%). Biogas upgrading is essential in order to increase the CH_4_ content, and thus its heating value, or when there is a provision for injecting biomethane into the natural gas network.^1^ As of 2016, there were 428 biomethane production plants in Europe using water-scrubbing (35.5%), chemical-scrubbing (20.5%), gas-separation membrane (20.5%), PSA (17%), organic-physical-scrubbing (4.5%), and other technologies,^2^ while by the end of 2021, there were 1067 biomethane plants (out of 18,843 biogas plants), aggregating to 37 TWh of biomethane or 3.5 bcm in Europe.^3^ A significant acceleration of the EU biomethane production by 2030 is also imperative to reach the target of 35 bcm as outlined in the REPowerEU Action Plan and its progress assessment.^4^

CO_2_ removal using membrane-based gas absorption and reaction is gaining attention as an alternative to well-established industrial gas-separation and reaction equipment, such as reactive-absorption columns, because of the high mass transfer area (known *a priori*) of the membrane modules per unit volume, their modular design, and easy scale-up of the process.^5−9^ A membrane bundle containing hundreds of fibers is placed within the membrane module with a customizable length, and it may provide a modular solution for easy lab- and pilot-testing with a specific surface area in the range of 1500–7000 m^2^/m^3 ^^10^ compared to 100–800 m^2^/m^3^ for conventional devices.^11^

Although the integration of the membrane into an intensified process offers many advantages modeling-wise, such as by distinguishing computationally the gas and liquid flows by distinct conservation equations in the two compartments, fiber and shell, with a known contact area, it also introduces a degree of uncertainty due to liquid intrusion into the membrane porous network, considerably diminishing the separation performance of the membrane contactor.^12^ Extensive reviews on membrane wetting can be found in Pantoleontos et al.,^8^ Mavroudi et al.,^13^ and Ibrahim et al.,^14^ where a description of this phenomenon is attempted along with possible causes and favorable conditions; below, references are updated and annotated in order to enunciate the arguments and the motivation of the current study.

The notion of partially wetted membrane pores stems from the discrepancy between the observed experimental values and the hitherto developed mass transfer models and correlations.^15^ The resistance-in-series approach is the standard method to analyze mass transfer through the membrane pores, which consists of the resistance of the gas-filled and the liquid-filled parts of the membrane pores as follows^8,13,16−23^
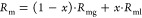
1where *R*_m_ is the total membrane resistance, *x* is the extent of wetting ranging from zero to one (continuous variable), and *R*_mg_ and *R*_ml_ are the individual resistances of the gas-filled and liquid-filled pores, respectively (to be analyzed in Section 3.2). Equation 1 is a linear expression with respect to the wetting extent and yields a straight line with a (positive) slope (*R*_ml_ – *R*_mg_) for constants *R*_mg_ and *R*_ml_, that is, for constant temperature and pressure when considering a binary mixture, provided there is no enhancement due to reaction in the liquid-filled part of the pores. In the corresponding literature, the extent of wetting, *x*, has been used to adapt computational models to experimental data; see, e.g., refs (8,14,16−18,22,24−26).

It is apparent from eq 1 that the resistances are added sequentially, assuming that gas first diffuses through the gas-filled part of the pore and then through the liquid-filled part of the pore due to any liquid penetration.^8^ Additionally, this approach renders the wetting extent a macroscopic averaged parameter of the overall porous network not considering any range of pore sizes, which would otherwise require knowledge of the pore-size distribution beforehand with all of the associated numerical difficulties (e.g., the type of distribution, the mean and maximum pore radius, the standard deviation, etc.^8^). While the series model combined with a single membrane pore-size value gives rise to a harmonic mean of the membrane mass transfer coefficients of the gas-filled and the liquid-filled part of the pores according to the extent of wetting (see Section 3.2), different approaches may hold, such as the parallel model leading to an arithmetic mean of the membrane mass transfer coefficients^23^ or a variation of the series model, treatments that are not unusual in porous media.

Relating the membrane module hydrodynamics with the wetting extent might be the ultimate target of a theoretical and modeling study, that is, investigating the effect of the gas and liquid flow rates on the membrane module performance with respect to membrane wetting. Hints on the liquid flow correlation with the wetting as predicted by eq 1 can be found in refs (20,21,27−30); in these studies with gas or liquid on the lumen side, the Lévêque correlation or the generalized Kreulen et al.^31^ or the Graetz-Lévêque correlation are used. These analytical solutions presuppose a Dirichlet boundary condition (BC) by applying equal concentrations at the outer parts of the membrane, for which, in principle and by definition, *no membrane mass transfer resistance* is assumed. Eloquently, any limiting parameter in the membrane to slow down mass transfer, such as wetting, is implacably inconsistent with the Lévêque correlation(s) application.^8,9,15^ This contradiction has not raised concerns about preventing other researchers from using the Lévêque correlation and its extensions (Kreulen,^31^ McAdams,^32^ Newman^33^) in combination with membrane wetting in general (see the review by Pantoleontos et al.^9^).

A recent account of the mass transfer behavior in a hollow fiber membrane contactor for CO_2_ absorption using tertiary amine solutions is presented by Yin et al.,^34^ who placed the solvent in the lumen side and the gas mixture in the shell. The authors showed that the computational wetting to match the experimental data decreases with the increase of the fiber-side liquid velocity, which indicates that an inverse relationship may exist between the liquid flow rate and the wetting, as also demonstrated by Pantoleontos et al. in a previous study when considering a physical-absorption case.^8^

The current study improves upon the previous work by Pantoleontos et al., who presented a CO_2_-in-water physical-absorption case along with macroscopic mass balance on the shell side, also accounting for process conditions, membrane and fluid properties, and module geometric characteristics.^8^ The ubiquitous resistance-in-series approach, as shown in eq 1, is a pertinent prerequisite in the analysis attempting to interpret the underlying wetting phenomena and aspiring to discern any wetting pattern. In this study, the shell-side mass balance equations include convective flux terms and a third-order reaction between CO_2_ and DEA, which enhances the overall mass transfer. The novelty of introducing a varying-with-length combined mass transfer coefficient is a key element that delivers a broad and rather unrestrained description of the overall model and the shell computational compartment. Parametric analyses of the shell-side mass transfer coefficient and the liquid-filled part of the membrane pore resistance are also attempted in order to assess different mass transfer regimes. Apart from the literature review in this section and in the Supporting Information file, references are incorporated and discussed throughout the remaining text for comparison, so that the mass transfer model along with transport resistances in each computational compartment is manifested in association with the terminology and assumptions of the model.

## 2 Experimental Section

**Figure 1:**
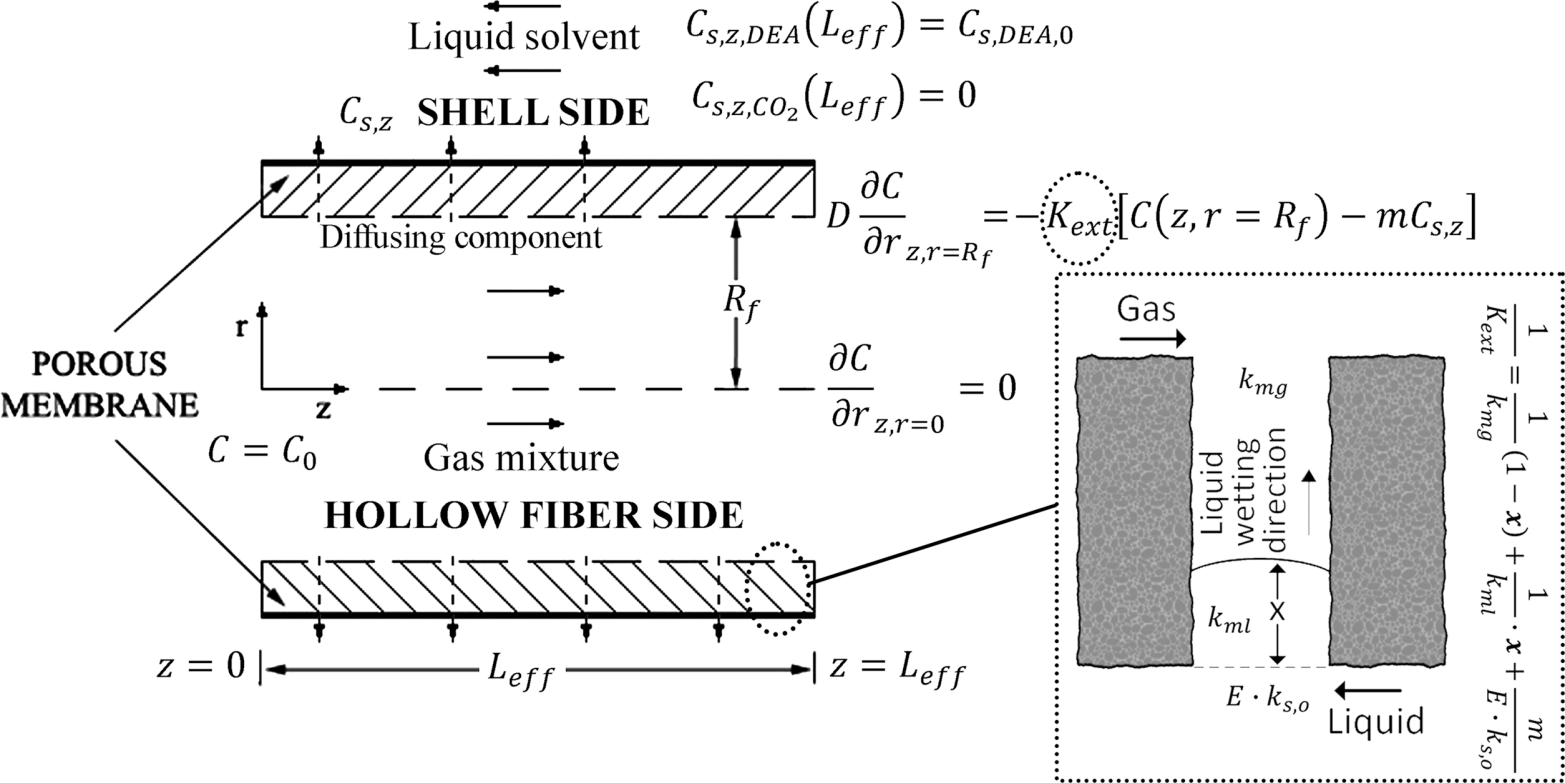
Single hollow fiber flow pattern with a gaseous mixture flowing in the lumen-side and countercurrent mode of operation depicting the corresponding boundary conditions along with the overall resistance (1/*K*_ext_) resolution into its constituent parts (adapted and reprinted from Mavroudi et al.,^17^ with permission from Elsevier).

The postulation of the flowing behavior is depicted in Figure 1, where the biogas mixture flows in the fiber side (lumen) and (mainly) CO_2_ diffuses through the membrane pores to be absorbed and to react with the medium flowing outside of the fibers, on the shell side, in the countercurrent mode of operation. Although the advantage of the counter mode over the cocurrent mode of operation is hardly new to process engineers, the interested reader may resort to Qin and Cabral^35^ and Pantoleontos et al.^8^ for theoretical aspects as well as the limitations of the cocurrent mode of operation. In the same figure, the individual mass transfer resistances are visualized as indicated in eq 1 and are further analyzed in subsequent sections including shell-side resistances. The piping and instrumentation diagram of the experimental gas–liquid contact membrane process unit has been illustrated by Asimakopoulou et al.^36^ and is included in the Supporting Information file, Appendix C.

Larger membrane modules (e.g., extraflow, EXF, in 3 M Liqui-Cel notation) provide enhanced mass transfer performance due to the presence of a center baffle directing “liquid radially across the membrane array” (on the shell side), capable of adding gases to or removing dissolved gases from compatible liquid streams.^37,38^ Thus, for the mini-module (MM) used in the current study (Liqui-Cel MM-1.7 × 5.5, Table 1), the same mode of operation is retained (gas through the fibers, liquid through the shell side) so that the main conclusions inferred from the experimental-modeling study are consistent with and may directly apply to the larger modules, provided the same postulation is applicable (2-D formulation in the fiber, 1-D in the shell), expectantly by altering the shell-side mass transfer coefficients.

**Table 1:** Gas-Liquid 1.7 × 5.5 Membrane Contactor Features and Their Notation in the Equations

commercial contactor type	3 M mini-module 1.7 × 5.5
cartridge configuration	parallel^38^
maximum flow rates (mL/min)	2500^38^
membrane	X50^38^
module length (mm)	182^38^
porosity of the membrane (ε_f_)	40%^38^
tortuosity (τ)	2.7^39^
pore size (μm) (2*R_p_)	0.04^39^
OD/ID (μm) (*d*_f,o_/*d*_f,i_)	300/220^39^
module diameter (mm) (*d*_m_)	42.5^39^
no. of fibers (*N*_f_)	7400^39^
effective fiber length (mm) (*L*_eff_)	113^39^
packing fraction (φ)	0.37^39^
active surface area (m^2^)	ID: 0.58

Aqueous solutions of MEA, DEA, and MDEA comprise the preferred amine solvents in conventional packed towers for CO_2_ capture.^29^ In the current study, during initial screening tests, MEA (Merck, ethanolamine for synthesis, purity >99%) and MDEA (Sigma-Aldrich, *N*-methyldiethanolamine ≥99%) apart from DEA (CHEM-LAB, v.p. for laboratory use, purity >99%) have been used. MDEA has a smaller capacity to react with CO_2_ compared with DEA. In the past, the process performance of MEA, DEA, and AMP was evaluated and compared in a 90% carbon-capture process from a 550 MW coal-fired power plant; from this, DEA was found to be a better performing solvent than MEA under optimized specifications in terms of energy penalty and capital cost investment.^40^ In addition, in the current study, the results showed that when an aqueous solution of MEA was used, a major deterioration in the mass flux through the wetted pores and an irreversible behavior of the membrane module even after drying overnight were observed. This behavior has also been indicated by Yeon et al., who observed that the removal efficiency of the MEA solution decreased to and remained constant at 75% after 8 h of operation due to wetting (compared to triethanolamine (TEA), which was stable) using PVDF membranes,^41^ or by Xu et al., who observed a dramatic flux drop of 71–89% in the CO_2_-MEA system using PP membranes throughout a 12-day operation (flux decline was also observed for PVDF membranes);^42^ see also deMontigny et al., who reported that PP membranes suffer a loss in performance when used with MEA solutions.^43^ Altering the solvent concentration may affect wetting;^14^ in all experiments, a constant molarity of DEA in aqueous solutions is maintained at 0.25 mol/L (0.25 M = 250 mol/m^3^), so that the calculated wetting values do not depend on the type or the concentration of the amine but only on the gas and liquid flow rates.

Regarding the membrane module, fittings and connecting hoses have been placed on every side of the membrane module (i.e., lumen and shell-side entry and exit). Pressure-regulating valves were used at the outlets of both gas and liquid phases in order to control the pressures of the two flows, while gas and liquid pressures were continuously monitored with pressure gauges on each side of the membrane module (i.e., entry and exit); the liquid pressure was regulated to be 0.1–0.5 bar higher than the gas pressure to prevent gas dispersion into the liquid phase. In the residue/analysis section, the liquid phase effluent was collected in the product barrel, and the treated gas was sent for online composition analysis (Hubei Cubic-Ruiyi Instruments CO. Ltd. Gasboard-3200 Online Biogas Analyzer; range: CO_2_: 0–50%; CH_4_: 0–100%; H_2_S: 0–9999 ppm; O_2_: 0–25%; accuracy: CO_2_, CH_4_: ≤ 1%FS; H_2_S, O_2_: ≤ 2%FS) and in the flowmeter (RITTER GAS METER TG1/5; 2–120 L/h; accuracy: ≤ ± 0.5% FS), after going through a water trap to protect the equipment. Each experiment was continually monitored and its conditions retained for almost 30 min of operation to ensure that steady state was reached. Indicative repetitive measurements were conducted for several data points, demonstrating high repeatability in the test results in all cases (relative differences <5% between the measurements). The membrane module was not dried afterward; small amounts of pure water were directed to pass through the module to clean any residual amine left on the shell side.

## 3 Model Formulation

Hollow fiber membrane processes have been mainly described by mathematical models involving concentration gradients within the fiber by solving the mass continuity equation.^9^ Similarities in the flow mode depicted in Figure 1 can be found in the work by Lu et al., who mainly used MDEA as the chemical solvent without considering membrane wetting or varying with the axial direction mass transfer coefficients.^44^ As there may be considerable concentration gradients on the shell side due to the presence of reactions, a mass balance on the shell side is crucial so that the driving force (concentration difference) between the lumen and the shell side is accurately defined.

The boundary conditions in the two computational departments (fiber and shell) are depicted in Figure 1. First, the countercurrent mode of operation renders the model a boundary-value problem with respect to the axial dimension since the conditions of the dependent variables are specified at the two extremes, *z* = 0 and *z* = *L*_eff_. Second, the solvent on the shell side enters at *z* = *L*_eff_ with its initial (feeding) concentration without any dissolved or reacted CO_2_. The two compartments are computationally coupled by the lumen-wall BC, which accounts for membrane and shell-side mass transfer resistances.

### 3.1 Fiber Side

The following assumptions are made to describe the fluid (gas) flow within the fiber and the associated BCs: (a) isothermal operation; (b) Newtonian fluid physical properties; (c) fully developed laminar flow in the lumen (fiber), with a parabolic velocity profile; (d) no homogeneous reaction in the fiber; (e) applicability of Henry’s law; (f) when the velocity profile is fully developed, the velocity term in the radial direction becomes zero;^9^ and (g) axial molecular diffusion can be neglected compared to axial convection when the axial Peclet number is greater than 100^9^ (valid in this study since the axial *Pe* number is >500).

Considering the assumptions above, the continuity equation in a fiber (lumen side) for every component, *i*, becomes
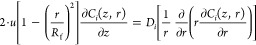
2with the associated boundary conditions (see also Figure 1)
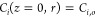
3
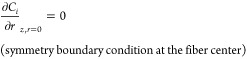
4
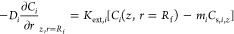
5where *u* is the average velocity in the lumen; *C*_*i*_ is the concentration of component *i* = CO_2_, CH_4_; *C*_*i*,o_ is the feed concentration of component *i*; *C*_s,*i*,*z*_ is the concentration of the diffusing component *i* on the shell side; *m*_*i*_ is the dimensionless partition coefficient (see Section 3.2) of the diffusing component *i* on the shell side; and *D*_*i*_ is the diffusion coefficient of component *i* (note that for a binary mixture as in this study, the diffusion coefficient is the binary diffusivity at the limit of infinite dilution and is thus independent of the concentration). The diffusion coefficients either are measured experimentally^45^ or can be calculated using molecular simulation approaches.^46−48^ A systematic methodology to this purpose has been discussed in detail in ref (49).

In principle, one has to solve the momentum transport equation in order to derive the axial velocity term, *u*_*z*_, especially for cases when there is significant loss of volumetric flow along the fiber due to the large diffusion of CO_2_ through the pores and the large contribution of CO_2_ in the biogas mixture. In order to alleviate this difficulty, the average axial velocity, *u*, takes into account the feed and the exit volumetric flow rate of the mixture, *Q*_g,in_ and *Q*_g,out_, respectively (as measured by the flow measurement analyzer; see Section 2), by introducing an average mixture flow rate, *Q*_g,avg_, as in eqs (S.3) and (S.4), Supporting Information file, Appendix A.

Equation 5 is a mixed Neumann–Dirichlet BC, which assumes that the mass flux at *r* = *R*_f_ is equal to the linear form of the combined mass transfer coefficient multiplied by the concentration difference of each component at the outer parts of the membrane; it also implicitly determines the flow along the fibers.^9^ The combined mass transfer coefficient is further analyzed in Section 3.2. A compendium of nonlinear lumen-wall mixed Neumann–Dirichlet BCs and their solutions most suitable for membrane processes such as supported liquid membrane, facilitated transport, membrane extraction, and others can be found in refs (6,9).

Note that the set of eqs 2–5 under steady-state conditions and no mass balance equations on the shell side (i.e., constant shell-side concentration) can be solved by the separation-of-variables method, yielding an analytical infinite-series solution, for which the computational code in commercial^6^ and open-source^9^ software is available. For very large values of the combined mass transfer coefficient, *K*_ext_, the lumen-wall BC is reduced to the prosaic Dirichlet BC and the associated entrance-region Lévêque–McAdams–Newman solutions (see the extended discussion in Pantoleontos et al.^9^ and references mentioned therein), whose dependent variable value is also predicted by the analytical solution when applying zero wall transport resistance.^6,9^ Furthermore, the presence of a liquid on the lumen side (unlike the present study; see Section 2), along with the rather indolent no wall-mass-transfer-resistance hypothesis, would postulate a model as applicable to entrance-region solutions (high Graetz numbers–Lévêque–McAdams–Newman correlations)^9^ with one important difference: a gas–liquid reaction case *in the lumen* should incorporate a reactive term in eq 2—a crucial deviation from the Graetz problem and the Lévêque solution(s).^9,50^ This element had escaped the attention of numerous authors in their modeling analysis when using the Lévêque correlation under reactive conditions on the lumen side.^19,21,29,30^

### 3.2 Membrane and Shell Side

The term *m* in eq 5 is the partition coefficient of each diffusing component corresponding to equilibrium conditions between the lumen and shell fluids depending on the physical properties of the fluids.^6,17,35^ The relationship between the gas–solvent dimensionless partition coefficient, *m*_A_, and Henry’s constant, *H*_A,solv_, for a solute is given by the following expression^9^
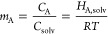
6where *C*_A_ is the concentration of gas A in the gaseous mixture, *C*_solv_ is the concentration of gas A in the solvent, *R* is the gas constant (Pa·m^3^/mol/K), and *T* is temperature (K). In the Supporting Information file, Appendix B, the relevant Henry’s constants for the system considered are presented.

For a case in which the gaseous mixture flows in the lumen and the species of interest diffuses through the membrane pores and reacts with the liquid on the shell side, the combined or external mass transfer coefficient, *K*_ext_, includes all relevant mass transfer resistances and layers of transport between the membrane and the shell-side boundary layer, *from where the lumen mass transport resistances end*:^9^ the membrane itself, including any partial wetting imposed by the penetrating liquid into the membrane pores, and the shell-side concentration boundary layer including any enhancement factor because of the reactive mixture
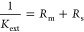
7where *R*_m_ and *R*_s_ are the membrane and the shell-side mass transfer resistances, respectively. The resistance-in-series model based on the two-film model for a gas–liquid interface as adopted in the present study is clarified by Danckwerts^51^ (see also references in this section and in Section 3.3). Equation 7 is obeyed strictly only under steady-state conditions; another prerequisite is that the molar flux through the different “layers” of transport (gas, membrane, liquid) should be the same.^52^ This formulation is somewhat different from the equality of molar flow rates followed by other researchers (see, e.g., ref (53)). Thus, in order for eq 7 to hold, it is convenient that a reference surface contact area common to all transport layers is defined.

Following eq 1, the membrane resistance has to be resolved into its constituent parts accounting for the extent of wetting, the gas-filled portion, *R*_mg,*i*,eff_, and the liquid-filled part of the pore resistance, *R*_ml,i,eff_, for every component *i*
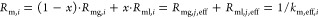
8where
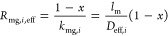
9
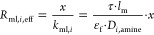
10where *k*_mg,*i*_ and *k*_ml,*i*_ are the membrane mass transfer coefficients if the whole length of the pore is filled with either gas or liquid, respectively; *k*_m,eff,*i*_ is the overall membrane mass transfer coefficient; *l*_m_ is the membrane thickness (as derived from the OD/ID entries in Table 1); *D*_eff,*i*_ is the membrane effective gas diffusivity (already including membrane porosity, ε_f_, and tortuosity, τ; see eq (S.12), Supporting Information file, Appendix B, and Table 1); and *D*_*i*,amine_ is the solute diffusivity into the liquid solvent (i.e., aqueous solution of DEA) for every component, *i*.

The concept of the film model is evident in eqs 9 and 10, as denoted by the definition of the mass transfer coefficient (inverse resistance) being equal to the diffusivity divided by a characteristic length. This is a critical statement that not only binds all relevant theoretical notions to the film-model paradigm (e.g., the enhancement factor) but also implies that, for instance, the transfer coefficient varies as *D*_A_ (cf. the surface renewal models, which predict that it varies as , among other differences). Eloquently, equally important from a practical point of view is the calculation of the diffusivities of the components in the gas and especially in the liquid solvent, as well as the assignment of the effective properties of the porous network.

For any reaction between a gas and a liquid to take place, the gaseous component must first diffuse and dissolve into the liquid. Its extent depends on the physical properties of the system (diffusivity, solubility), the reactive conditions (intrinsic kinetics, which is the same for all types of reactors), and the process itself, which determines the apparent (observable) mass transfer coefficient.

The general expression for the shell-side resistance is given by^35^
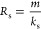
11where *k*_s_ is an apparent mass transfer coefficient accounting for reactive conditions, that is
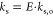
12

In other words, the apparent shell-side mass transfer coefficient is derived by multiplying E-times (*E* > 1) the mass transfer coefficient, *k*_s,o_, with the latter alluding to physical absorption. Obviously, in the absence of reaction, *E* = 1 (e.g., for CH_4_ dissolving in the shell-side solvent). Expressions and approximations for calculating the enhancement factor for irreversible *m*th- and *n*th-order reactions are given in Section 3.3. Note that unlike previous modeling attempts, in the current study, the apparent shell-side mass transfer coefficient, *k*_s_, the combined mass transfer coefficient, *K*_ext_, and the enhancement factor, *E*, vary with the *z* direction.

In principle, since in the liquid-filled part of the membrane pores there is contact between the gas and the liquid, the total membrane mass transfer coefficient has to account for the enhanced mass transfer due to reactive conditions.^54,55^ Nevertheless, the enhancement might be dissimilar due to the different mass transfer mechanisms in the membrane and in the liquid film on the shell side.^54^ An extreme case is also added in the corresponding analysis by solving the set of equations using an altered version of eq 10 by including an averaged enhancement factor, *E*_avg_, over the reaction path (i.e., over the axial length of the computational domain); see also the next section
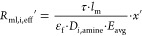
13

The shell-side mass transfer coefficient, *k*_s,o_, is provided by correlations depending on the configuration of the module (i.e., parallel flow or cross-flow). In parallel flow, the value of the coefficient may be higher than 10^–4^ m/s, having a difference between 1 and 3 orders of magnitude (see the review by Estay et al.^56^). A relevant correlation for parallel flow is given by Prasad and Sirkar^53^
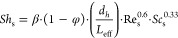
14where φ is the packing fraction of the bundle of fibers in the module (given in Table 1).

Yang and Cussler, who also derived a shell-side correlation, reckon that a high shell-side void volume might amplify bypassing and channeling on the shell side,^52^ a possibility that is also demonstrated by Basu et al., who measured shell-side Sherwood numbers 3 to 3.5 higher than those predicted by the original correlation by Prasad and Sirkar when packing densities ≥0.4 were utilized.^57^ Yun et al. set a value of β = 17.4,^58^ which adjustably increases the physical-absorption rate.

The Yang and Cussler correlation is defined as^52^
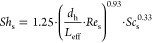
15

Another parallel-flow shell-side correlation has been developed by Costello et al.^59^
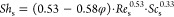
16

It may well be reserved that the shell-side correlations developed are module-specific (e.g., applicable to the module configuration, the packing fraction, the hollow fiber type, the dimensions of the module, etc.) and process-specific (e.g., solvent extraction or gas absorption, accounting for the phase of the fluids circulating in the module), giving rise to ambiguity on the design and performance assessment.^56^ Due to the high uncertainty of the shell-side mass transfer correlation, for the current study purposes, all mentioned shell-side correlations (eqs 14 – with values by Yun et al.), 15 and 16, have been used, which also serve as a parametric analysis to examine the effect on mass transfer and computational wetting, a vital analysis for interpreting the results and the concluding observations.

By definition, the shell-side dimensionless numbers, namely, the Sherwood number, *Sh*_s_, the Reynolds number, *Re*_s_, and the Schmidt number, *Sc*_s_, are given by the following expressions (for CO_2_; similarly for CH_4_)
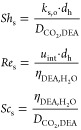
17

Steady-state convective transport on the shell side accounting for reactive conditions and countercurrent mode of operation for the species of interest can be written as
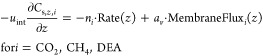
18
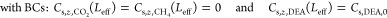
19where MembraneFlux_*i*_(*z*) = *K*_ext,*i*_[*C*_*i*_(*z*, *r* = *R*_f_) – *mC*_*s*,*i*,*z*_] (zero for DEA), Rate(*z*) (zero for CH_4_) is defined in Section 3.3, *n*_*i*_ is the species stoichiometric coefficient (2 for DEA; 1 for CO_2_), and *C*_s,DEA,0_ is the feed molarity of DEA. The terms *a*_*v*_ and *u*_int_ are discussed in the Supporting Information file, Appendix A.

### 3.3 Reaction and Enhancement Factors

It is important to appreciate that the concentration of the diffusing component on the shell side, *C*_s,*z*_, refers to the component itself and not to any other species related to this component, e.g., an ion formed in a reactive mixture. In other words, for a reactive case that results in the reaction of the original component forming another species, the concentrations of the set of eqs 2–5, 18, and19 include only the unreacted amount on the shell side; ion species concentrations are inferred from the extents of the (independent) reactions.

A general rational expression for the reaction rate between CO_2_ and DEA according to the zwitterion reaction mechanism (see also the Supporting Information file, Appendix C), which is valid for a wide range of DEA molarities, is proposed by Versteeg and van Swaaij,^60^ with parameters taken from the work by Versteeg and Oyevaar.^61^ Hikita et al. presented reaction kinetics between three alkanolamines (MEA, DEA, and TEA), especially for mild concentrations of DEA (0.174–0.719 M) and varying temperature (5.8–40.3 °C). The reaction rate, Rate, is expressed as an overall third-order reaction^62^
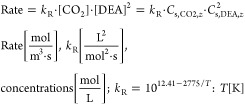
20

In order to simplify the complexity of the enhancement factors’ expressions as implied from the use of a rational rate function due to Versteeg et al.^60,61^ (see, for example, the methodology by Onda et al.^63^), in the current study, a reaction rate based on the overall reaction for DEA
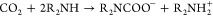
21and the reaction rate expression by Hikita et al. are used with R = CH_2_CH_2_OH^–^ for DEA.

It has to be noted that in this study, as reaction 21 indicates, an irreversible reaction is assumed, a common treatment in similar research studies.^44,64^ An elegant description of both forward and reverse reaction rates is presented by Delgado et al.,^39^ whose kinetic parameters for discrete points of DEA and CO_2_ concentrations are provided with the aid of literature values and equilibrium speciation modules in an Aspen Plus process modeling environment without needing enhancement factors.

For the general case of an irreversible *p*th-, *q*th-order reaction (i.e., *r* = *k*_pq_*a*^p^*b*^q^), approximate expressions for calculating the enhancement factor are given by^51,65^
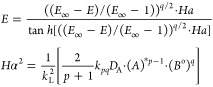
22where the asymptotic infinite enhancement factor is given by^64^
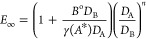
23where *B*^o^ is the concentration of free (unreacted) amine, (A*) is taken as the concentration of the dissolved solute multiplied with *m* as in eq 24, *Ha* is the Hatta number, γ is the stoichiometric coefficient of reactant *B*, *k*_L_ is the physical-absorption mass transfer coefficient, and *n* = 0 for the film model and *n* = 1/2 for the penetration model.^64^ In the present study, in order to be consistent with the resistance-in-series film model, *n* = 0. Following the third reaction rate expression (21) for the system CO_2_-DEA (i.e., *p* = 1, *q* = 2) and the nomenclature of the Supporting Information file, Appendix A, eqs 22–23 become
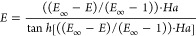
24
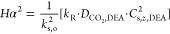
25
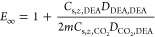
26

**Figure 2:**
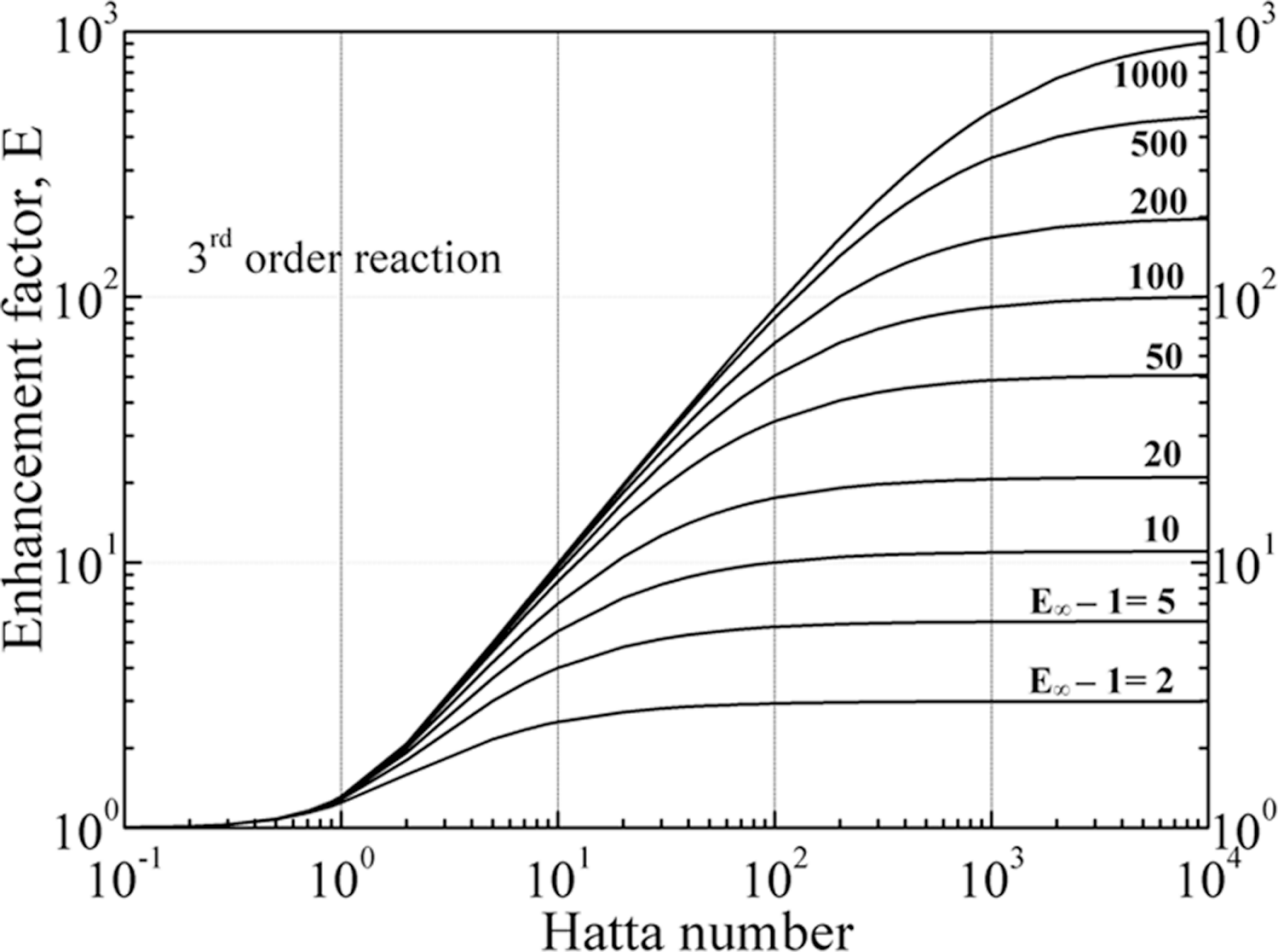
Enhancement factors for third-order reaction.

Figure 2 depicts the relation of the enhancement factor, *E*, with the Hatta number, *Ha*, for various (*E*_∞_ – 1) values and a third-order reaction according to eq 24. Since *E* could never exceed the *E*_∞_ value, it asymptotically converges to the infinite enhancement value for large Hatta numbers. When the Hatta number is much smaller than unity, then *E* ≈ 1 regardless of the value of *E*_∞_; when *Ha* ≤ 1, then *E* > *Ha*; for higher Hatta values, the influence of *E*_∞_ becomes significant. It has to be noted that Figure 2 illustrates an almost identical behavior to a second-order reaction (cf. Danckwerts^51^), which is also straightforward to implement using the expressions eqs 22 and 23 for irreversible reactions.

There is a notable region in which *E* ≈ *Ha* (when *E*_∞_ is much higher than *Ha* and *Ha* > 1); in such a situation, eq 12 becomes [according to eq 25]
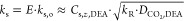
27which is a key element in the subsequent analysis of the current study, entailing that the apparent mass transfer coefficient, *k*_*s*_, would depend mainly on the reactive and not on the flow conditions (independent of *k*_s,o_) on the shell side; note that working in this regime favors the determination of intrinsic kinetics of chemical reactions.^66^

Equations 24–26 imply that all dependent variables, *E*, *E*_∞_, and *Ha*, vary along the path of reaction since the concentrations of both CO_2_ and DEA are not constant. An averaged enhancement factor introduced in the previous section for an altered version of the liquid-filled part of the membrane pores, eq 13, is defined as
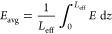
28

This averaged quantity is in accordance with the current model use of mean parameters in the overall porous network, such as the wetting extent, the tortuosity and porosity, and the mean pore size as provided by the manufacturer. In Section 4.2, it is shown that the mass transfer behavior is better described by eq 10 rather than eq 13 by comparing with a physical-absorption case.

Summing up the analysis in this section, the reaction rate expression is crucial in order to determine the consumption and formation of various species on the shell side, whose variation can be predicted by eqs 18 and 20 or can be inferred from the stoichiometry of the reaction; however, the enhancement factors are used only for the determination of the apparent shell-side mass transfer coefficient (as applied in the shell-side boundary layer), *k*_s_.

### 3.4 Method of Solution and Some Remarks

Unlike past studies where dimensionless quantities have been presented to derive a dimensionless system of equations,^6,8,9^ such as the dimensionless concentration and the Graetz number (see the Supporting Information file, Appendix A), in this study, the dimensional PDE system of eqs 2–27 is solved. The incorporation of the inverse Graetz numbers as the dimensionless axial codomain requires updated discretization with respect to the axial direction every time different gas flow rates are introduced. For further reading about the traditional dimensionless Graetz problem for various lumen-wall BCs, the reader is referenced to Pantoleontos et al.;^6,8,9^ see also Section 4 for further analysis.

The boundary-value problem emerging from the countercurrent mode of operation necessitates that discretization in both (z and r) directions be applied, which leads to a system of N × M algebraic equations under steady-state conditions (N and M refer to the discretization r- and z-node points, respectively).

In order to calculate the combined shell and membrane mass transfer coefficient, *K*_ext_, it is necessary to know the extent of wetting, x. Since only one parameter is missing from the overall system of equations, a procedure matching the experimental data with the computational results is employed, rendering the problem a simulation procedure (rather than an optimization one) by setting^8^
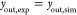
29where *y*_out_ is the molar fraction of CO_2_ at the exit of the lumen side for the measured (*exp*-subscript) and the model prediction (*sim*-subscript), with mixed-cup quantity.

The SPSE gPROMS ModelBuilder 7.1 process modeling environment^67^ is chosen for automatic discretization and simulation of the PDE system using the orthogonal collocation on finite element method with 64 collocation points in each (z, r) domain; these collocation points are concentrated with respect to the r-domain in the region near the membrane inner wall due to the sink-term BC (5) and at the exit of the fiber zone (due to the countercurrent mode of operation), where sharp gradients are expected. Alternatively, the user may specify nonuniform grid points in gPROMS for a normalized domain (i.e., the outer node points are fixed at 0 and 1 automatically); for example, a Gauss–Jacobi–Lobatto orthogonal-collocation scheme may be invoked externally to generate roots for the dimensionless r-domain.^9^ Another feature of gPROMS is that it can handle the implicit declaration of eq 24 with respect to *E* (normally, it should be solved numerically by iteration): gPROMS can handle this equation without user intervention/manipulation (i.e., just writing eq 24 as is).

SPSE gPROMS makes use of the BDNLSOL method, which stands for “Block Decomposition NonLinear SOLver”. As a modular solver component, BDNLSOL can, in principle, make use of any other nonlinear solver component to solve its individual blocks. A preset initial-guess matrix is imperative for fast and successful initialization and solution. Special care should be taken for variables having values less than the default precision/tolerance values set by gPROMS. Instead of regulating the *PROCESS* entity entries in the “Solution parameters” tab to very small values, it is recommended that, e.g., the gas–liquid diffusivities not be written in SI units (better: cm^2^/s) or a parameter (e.g., 10^6^) be multiplied with the original value. Nevertheless, the values of “Absolute tolerance”, “Event tolerance”, and “Relative tolerance” in the DASolver parameters could be set to 10^–7^ without encountering any problem; see also the corresponding help-pages of SPSE gPROMS.

## 4 Results and Discussion

In this section, the results derived in the combined experimental-simulation study are presented and discussed.

### 4.1 Absorption and Reaction of CO_2_ Using the Aqueous Solution of DEA: Experimental Results

Table 2 summarizes the conditions, the gas and liquid flow rates, and the feed (lumen entrance) and exit (lumen outlet) molar fractions of CO_2_ as experimentally measured during the experimental campaign conducted in the gas–liquid contact membrane unit with the 3 M Mini-Module 1.7 × 5.5 (see Table 1). Specifically, the gas mixture containing 41.2–41.9% CO_2_ and the remaining containing 58.8–58.1% CH_4_ enters the lumen-side (within the fibers; see Figure 1), diffuses through the membrane pores, and is dissolved and reacts with the solvent (aqueous solution of 0.25 MDEA) flowing counter-currently on the shell side of the membrane module. Gas flow rates vary from 2.84 × 10^–5^ to 3.63 × 10^–5^ m^3^/s (102 to 131 L/h), and the liquid flow rates vary from 0.47 × 10^–5^ to 0.58 × 10^–5^ (17 to 21 L/h), well within the operating guidelines for the specific module used;^38^ the intention is to present an adequate number of experimental data points of more than 65% and less than 100% CO_2_ removal for postprocessing analysis. Experiments are carried out at ambient temperatures from 298 to 302 K at atmospheric pressure, and the data are used in order to examine and validate the model. CH_4_ diffusion through the membrane into the liquid flow can be considered negligible in all experimental sets according to the experimental measurements and mass balance calculations. This is in accordance with McLeod et al., who also observed that CH_4_ slip into a sodium hydroxide solution can be preserved at 0.1% under chemical conditions.^68^

**Table 2:** Experimental Results for the System CO_2_-DEA (0.25 M) and Additional Dimensionless Numbers

no.	*T* (K)	Pg_in_ (atm)	Pg_out_ (atm)	Qg_in_ × 10^–5^ (m^3^/s)	Qg_out_ × 10^–5^ (m^3^/s)	*Q*_l_ × 10^–5^ (m^3^/s)	*y*_in_ (%) (CO_2_)	*y*_out_ (%) (CO_2_)	CO_2_ removal (%)	*ẑ*	m*
1	301.5	1.0036	1.0022	2.844	1.872	0.467	41.2	9.35	85.08	392.36	7.80
2	301.5	1.0036	1.0020	2.844	1.742	0.517	41.2	2.90	95.70	392.36	7.04
3	301.5	1.0036	1.0019	2.844	1.694	0.583	41.2	0.62	99.11	392.36	6.24
4	297.5	1.0043	1.0037	3.236	2.283	0.467	41.2	15.20	73.99	336.11	8.09
5	298.5	1.0046	1.0036	3.214	2.083	0.508	41.6	8.15	87.31	340.46	7.55
6	298.5	1.0046	1.0024	3.214	1.986	0.583	41.6	3.56	94.72	340.46	6.58
7	297.5	1.0051	1.0031	3.658	2.675	0.467	41.7	18.70	67.28	297.08	9.14
8	298.5	1.0050	1.0030	3.633	2.469	0.508	41.9	12.30	80.09	301.04	8.54
9	298.5	1.0050	1.0028	3.633	2.347	0.583	41.9	7.38	88.65	301.04	7.44

As seen from Table 2, the removal efficiency increases with the increase of the liquid flow rate (for a given gas flow rate) as higher amounts of liquid solvent are available to react with the gas diffusing from the lumen side through the membrane pores into the shell side. The experimental conditions and mode of operation justify the choice of the intensified absorption-reaction process using polypropylene membrane modules, achieving more than 67% removal of CO_2_ with a very low DEA concentration, while the removal efficiency exceeds 90% and may reach 100% for a gas-to-liquid flow rate ratio equal to 4.9 in the inlet (*Q*_g,in_/*Q*_l_). Thus, it can be concluded that the highest possible recovery of CH_4_ in a highly concentrated fiber exit stream can be achieved since very high removal of CO_2_ is maintained using a DEA solution of mild concentration.

The term m* (= *mQ*_g,in_/*Q*_l_), defined as the absorption factor,^35^ is the adjusted gas-to-liquid flow rate ratio accounting for the equilibrium coefficient, as denoted in eq (S.1), which along with the inverse Graetz number, *ẑ* (= 1/*Gz* = π·*N*_f_·*L*_eff_·*D*/(4*Q*_g,in_)), can predict the averaged and the overall lumen mass transfer coefficients for known *K*_ext_ values for the general case of nonconstant shell-side concentration of the species of interest (for their physical meaning and magnitude, see, e.g.,^6,8,9,35^). As part of the membrane modeling framework, these dimensionless terms determined by the fluid flow rates and their physical properties can be used for comparison purposes with other membrane-based systems.

**Figure 3:**
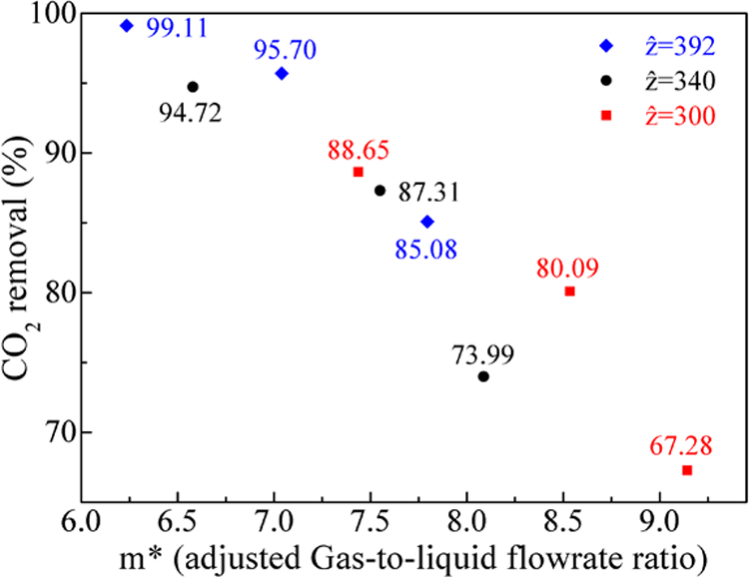
CO_2_ removal efficiency with respect to the adjusted gas-to-liquid flow rate ratio (m*) for three different *ẑ* (inverse Gz number) sets.

Figure 3 illustrates the findings of Table 2 regarding the CO_2_ removal efficiency with respect to m* for three different *ẑ* sets. (Note that these are reference values at the fiber inlet since, in principle, the gas flow rate varies within the lumen, a variation that has been taken into account in the current model for the calculation of the average gas mixture velocity; see the Supporting Information file, Appendix A.) It is shown that the higher the *ẑ* (the lower the Gz number), the higher the removal efficiency of the module.

A linear correlation with respect to the unknown parameters has been developed, interpolating CO_2_ removal with respect to *ẑ* and m*, according to the experimental data of Table 2, which is given by
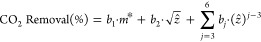
30

Statistical analysis and the calculated *b*_*j*_ coefficients are provided in Table 3. It has to be noted that this correlation (30) has no particular physical meaning, but rather it may serve for design since it describes very well the experimental data of Table 3; nevertheless, it is strictly valid for the biogas composition, the flow rate ranges chosen, the type of membrane module, and the concentration of DEA (0.25 M).

**Table 3:** Calculated b_j_ Coefficients and the Corresponding Regression Analysis

coefficients	values	stand. error	experimental no.	predicted CO_2_ removal (%)	residuals
*b*_1_	–8.3715 × 10°	1.2436 × 10°	1	86.84	–1.76
*b*_2_	6.3626 × 10^5^	1.6191 × 10^5^	2	93.16	2.54
*b*_3_	–3.6259 × 10^6^	9.2210 × 10^5^	3	99.89	–0.79
*b*_4_	–3.2680 × 10^4^	8.3216 × 10^3^	4	73.99	0.00
*b*_5_	3.2609 × 10^1^	8.3158 × 10°	5	86.96	0.36
*b*_6_	–1.9449 × 10^–2^	4.9677 × 10^–3^	6	95.08	–0.36
multiple R	0.994		7	67.28	0.00
*R*^2^	0.988		8	79.78	0.31
stand. error	1.881		9	88.96	–0.31

### 4.2 Membrane Wetting Estimation

In order to derive the relevant mass transfer coefficients (see Section 4.3), the system of equations in the dimensional form, including eqs 2–10, (S.4), (S.10), 18–19, 20, 24–26, 29, and depending on the shell-side mass transfer correlation, one of eqs 14 (with values by Yun et al.), 15, or 16 is solved. The incorporation of eq 29 conveniently obviates the need for iterative calculations since the matrix of algebraic equations is consistent, and the problem can be solved as a simulation rather than an optimization procedure. Apart from the feed conditions, two experimental quantities are needed in the computational model: the molar fraction of CO_2_ and the volumetric flow rate of the mixture at the exit of the membrane module (see also Sections 2, 1, and 3.4).

Table 4 presents the main findings in terms of the wetting values and other related variables of the simulation procedure, matching the experimental data of Table 2 (i.e., the “CO_2_ removal (%)” column) when the reaction rate by Hikita et al.^62^ in combination with either of Costello et al.,^59^ Yun et al.,^58^ or Yang and Cussler^52^ is taken into account. Apart from wetting values, which apply for both gases CH_4_ and CO_2_, as a metric of the extent of the solvent penetration into the membrane pores, all other values refer to CO_2_ and the interaction of CO_2_ with DEA.

**Table 4:** Simulation Results Regarding Shell-Side and Membrane Mass Transfer Coefficients Using the Hikita et al. Reaction Rate^62^ and the Associated Averaged Enhancement Factors

no.	*x* (wetting)^a^	*x* (wetting)^b^	*x* (wetting)^c^	*k*_s,o_ × 10^–5^ (m/s)^a^	*k*_s,o_ × 10^–6^ (m/s)^b^	*k*_s,o_ × 10^–7^ (m/s)^c^	*R*_ml_ × 10^5^ (s/m)	*R*_mg_ × 10 (s/m)	*k*_m,eff_ × 10^–4^ (m/s)^a^	*k*_m,eff_ × 10^–4^ (m/s)^b^	*E*_avg_^a^	*E*_avg_^b^	*x*′ (wetting)^d^	*x*′ (wetting)^e^
1	0.0737	0.0661	0.0384	1.683	2.863	6.760	1.292	6.905	1.044	1.163	21.10	104.85	1.5712	7.3386
2	0.0400	0.0335	0.0117	1.776	3.044	7.431	1.292	6.905	1.912	2.278	21.29	106.62	0.8605	3.7818
3	0.0222	0.0175	0.0037	1.894	3.274	8.319	1.292	6.905	3.414	4.290	21.31	109.04	0.4773	2.0279
4	0.0782	0.0685	0.0305	1.549	2.618	5.986	1.419	6.982	0.895	1.022	17.92	85.85	1.4139	6.1339
5	0.0480	0.0378	negative	1.655	2.819	6.684	1.386	6.963	1.488	1.885	18.09	85.12	0.8759	3.3600
6	0.0302	0.0225	negative	1.781	3.062	7.596	1.386	6.963	2.354	3.135	18.04	87.07	0.5489	2.0502
7	0.0803	0.0682	0.0210	1.549	2.618	5.986	1.419	6.983	0.873	1.026	17.09	79.19	1.3837	5.6182
8	0.0517	0.0381	negative	1.655	2.819	6.684	1.386	6.964	1.384	1.869	16.92	75.45	0.8813	2.9885
9	0.0352	0.0245	negative	1.781	3.062	7.596	1.386	6.964	2.024	2.888	16.75	76.40	0.5939	1.9449

a Costello et al.^59^

b Yun et al.^53,58^

c Yang and Cussler.^52^

d Costello et al.; use of eq 13.

e Yun et al.; use of eq 13.

The use of the Yang & Cussler correlation,^52^eq 15, does not yield results of physical meaning for some gas–liquid flow rates (i.e., resulting in negative wetting values). This occurs because this correlation predicts small mass transfer on the shell side (the order is Costello et al. > Yun et al. > Yang and Cussler; see Table 4), and in some cases, there is much smaller CO_2_ removal than the experimental results even with zero wetting value. On the other hand, the Hikita–Costello and the Hikita–Yun pairs yield wetting values within the range {0, 1}, at least for the flow rate ranges examined in this study. Thus, it may well be concluded that the overall model is essentially limited by the “choice” of the shell-side mass transfer correlation since it might yield unacceptable (by definition) wetting values.

**Figure 4:**
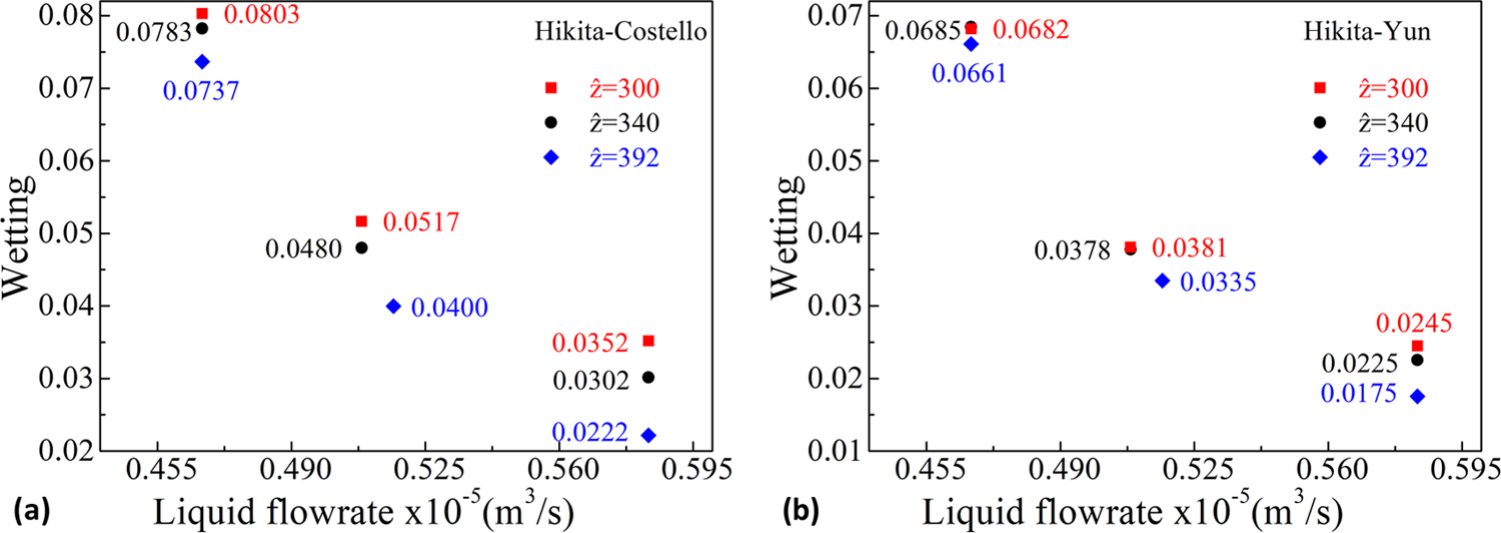
Membrane wetting with respect to the liquid flow rate for the three *ẑ* sets for the Hikita–Costello (a) and the Hikita–Yun (b) pairs without any enhancement in the liquid-filled part of the pores; eq 10 is active.

Figure 4 depicts the calculated wetting values (Table 4) for all experimental data for the Hikita–Costello and the Hikita–Yun pairs with respect to the liquid (shell-side) flow rate with the aid of groups in terms of the *ẑ* number. It is seen that the wetting values are concentrated close to each other mainly depending on the shell-side liquid flow rate (more so for the values calculated by the Yun et al. correlation, as listed in Table 4). In other words, the liquid velocity on the shell side largely determines the extent of liquid penetration into the membrane pores as would be expected,^69^ a decisive factor for the justification of the postulated model and its assumptions.

Nevertheless, the gas mixture flow rate (inversely proportional to *ẑ*) seems to also influence the wetting estimation, deriving smaller wetting values with higher *ẑ* values (smaller feed gas mixture velocities) when considering the use of the Hikita–Costello pair. Pantoleontos et al.^8^ and Yin et al.^34^ point toward an inverse relationship, i.e., a smaller feed gas mixture velocity for the same liquid flow rate yields higher computational wetting values. Presumably, the use of different correlations (cross-flow module shell-side correlation used by Pantoleontos et al.^8^), distribution of different gas–liquid flows (e.g., gas in the shell, liquid (and reaction) in the lumen in Yin et al.^34^), and the modifications of the current model (e.g., *K*_ext_ and enhancement factors varying with *z*) may also elicit different trends in the simulation results. Furthermore, it is shown that an implicit relationship*—*whatever that is*—*between the gas mixture flow rate and the computational wetting may exist, which cannot be suppressed by the hitherto developed model postulations. Still, the use of the Hikita–Yun correlation indicates that there is no discernible pattern or influence of the gas mixture velocity on the computational wetting; this observation indicates that this correlation is the most consistent with the perception of a liquid-only computational wetting source.

As seen in Figure 4, a wetting pattern can be discerned for both correlations with an increasing liquid flow rate leading to smaller wetting in the membrane pores, as also demonstrated in a previous work by Pantoleontos et al., who considered physical absorption of CO_2_ into water.^8^ The correlation between increasing liquid flow rates and lower wetting values does not imply that the liquid amount penetrating into the membrane pores diminishes in absolute terms, but rather that the relative liquid-part membrane resistance is lower than the gas-filled membrane resistance. Apparently, higher liquid charging on the shell side results in a higher shell-side velocity and smaller shell-side concentration boundary layer resistance, enhancing the diffusion of gaseous components into the liquid bulk.^8^ The same wetting pattern of an inverse relationship between the liquid flow rate and the wetting has also been implied by Yin et al. in their experimental-modeling study and the corresponding figures (experiments in a hollow fiber membrane contactor for CO_2_ absorption and interpretation with the aid of a fiber-membrane-shell model using tertiary amine solutions):^34^ that the computational wetting to match the experimental data should decrease with the increase of the fiber-side liquid velocity. (Additional graphs regarding the total membrane mass transfer resistance with respect to wetting values are provided in the Supporting Information file, Appendix C.)

**Figure 5:**
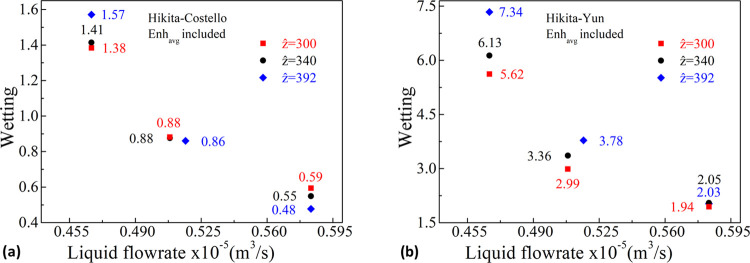
Membrane wetting with respect to the liquid flow rate for the three *ẑ* sets for the Hikita–Costello (a) and the Hikita–Yun (b) pairs with eq 13 activated—an extreme enhancement in the liquid-filled part of the pores.

For the sake of comparison, the simulation pairs of Hikita–Costello and Hikita–Yun are reconsidered including eq 13 instead of eq 10 as depicted in Figure 5. This is an extreme case assigning the entire value of the enhancement due to reaction in the shell-side liquid film to the liquid-filled part of the pores. This formulation is evidently wrong since it yields wetting values of no physical meaning (>1); they are not consistent with the physical-absorption values found by Pantoleontos et al. in the cross-flow configuration mode (maximum value equal to 0.21)^8^ or, for instance, by Yin et al., who calculated values ≤0.15 in reactive-absorption experiments.^34^ Inclusion of the averaged enhancement factor, *E*_avg_, in the equations of the liquid-filled part of the pores results in a substantial decrease of the corresponding mass transfer resistance and thus an increase (overestimation) of the computational wetting in order for the model predictions to match with the experimental data. Since the enhancement factor aligns with the shell-side mass transfer correlation value, indicating the increase in mass transfer for the reactive case compared with the physical one (see Table 4), its value is higher for the Yun et al. runs than those for the Costello et al. ones. Nevertheless, this extreme case does not necessarily exclude the possibility of a slight enhancement of mass transfer in the liquid-filled part of the membrane pores due to reaction. In the next companion section, mass transfer aspects are presented in order to further clarify relevant phenomena, where a unified approach is attempted.

### 4.3 Mass Transfer Analysis and Reaction Rates

**Figure 6:**
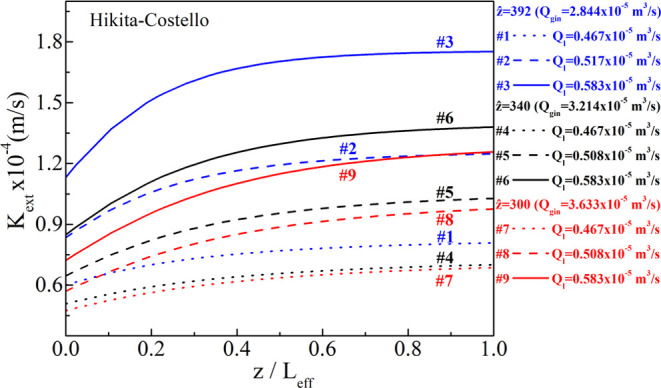
Variation of *K*_ext_ along dimensionless *z*/*L*_eff_ for all experimental sets for the Hikita–Costello pair.

**Figure 7:**
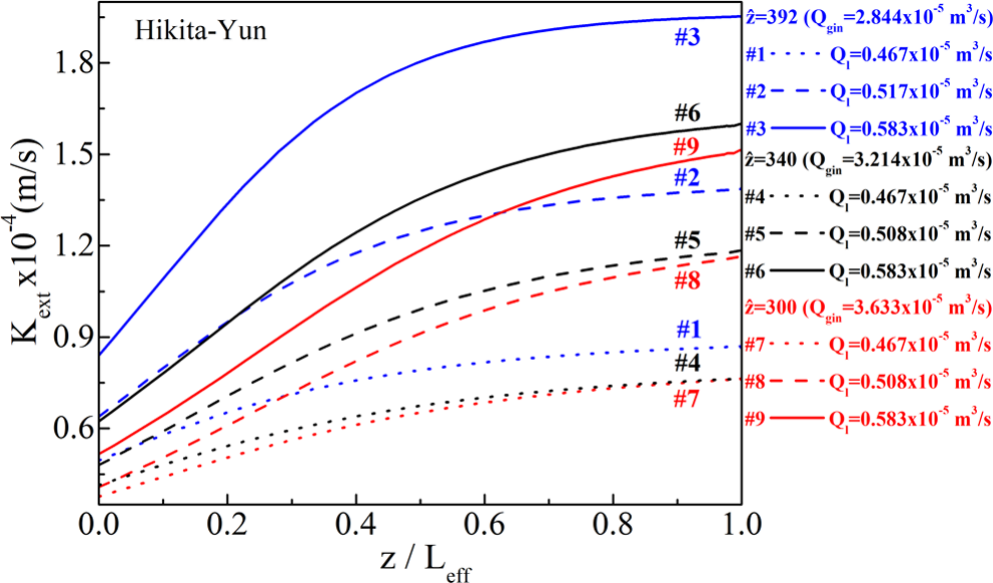
Variation of *K*_ext_ along dimensionless *z*/*L*_eff_ for all experimental sets for the Hikita–Yun pair.

Figures 6 and 7 depict the calculated variation of *K*_ext_ along the dimensionless *z*/*L*_eff_ (not equal to *ẑ*) for all (nine) experimental sets of Table 2 for the two shell-side mass transfer correlations (eq 14, with values by Yun et al., or 16). First, allowing the transfer coefficient to vary with length is an improved approximation of the mass transfer behavior, as evident in the figures, at least for the flow rates and the DEA concentration chosen, without encountering any numerical difficulties. For each *ẑ*-set (blue, black, or red), the highest *K*_ext_ values are found for the highest liquid flow rate from the set (e.g., for *ẑ* = 392 (blue set, #1, #2, #3), the highest *K*_ext_ values are for *Q*_l_ = 0.583 × 10^–5^ m^3^/s). For the same liquid flow rate (line style: solid, dash, or dot), the highest *K*_ext_ values are found for the lowest gas flow rates from the set (e.g., for *Q*_l_ = 0.583 × 10^–5^ m^3^/s (line style: solid, #3, #6, #9), the highest *K*_ext_ values are for the lowest *Q*_g,in_ = 2.844 × 10^–5^ m^3^/s), indicating that the same liquid flow rate can accommodate *better* less amounts of gas.

For the interpretation of the results, one has to comprehend the very definition of *K*_ext_ with the aid of Figure 1; the individual resistances that compound the combined mass transfer resistance commence from where the lumen mass transport resistances end,^9^ as already noted in Section 3.2; thus, its value is directly influenced by the phenomena occurring in the shell and in the membrane. Even when considering the overall mass transfer coefficient, the rationale remains the same: Nieminen et al., in membrane-based CO_2_ removal experiments in aqueous potassium glycinate, calculated higher mass transfer coefficients for the lowest CO_2_-feed composition (for the same gas mixture and liquid flow rates).^70^

Instructive results are observed for the same liquid flow rate (line style: solid, dash, or dot) when increasing the biogas flow rate (decrease of the inverse Graetz number, *ẑ*) for both shell-side correlations. For example, for *Q*_l_ = 0.467 × 10^–5^ m^3^/s (line style: dot, #1, #4, #7), increasing the gas flow rate from 3.214 × 10^–5^ to 3.633 × 10^–5^ m^3^/s becomes less efficient in terms of the combined mass transfer coefficient, *K*_ext_, yielding almost equal *K*_ext_ values for the #4 and #7 experimental sets. This implies that the mass flux through the membrane pores when further increasing the gas flow rate without increasing the liquid flow rate (or increasing the DEA molarity) will be driven merely by the concentration difference at the outer parts of the membrane without any substantial improvement due to the mass transfer coefficient.

**Figure 8:**
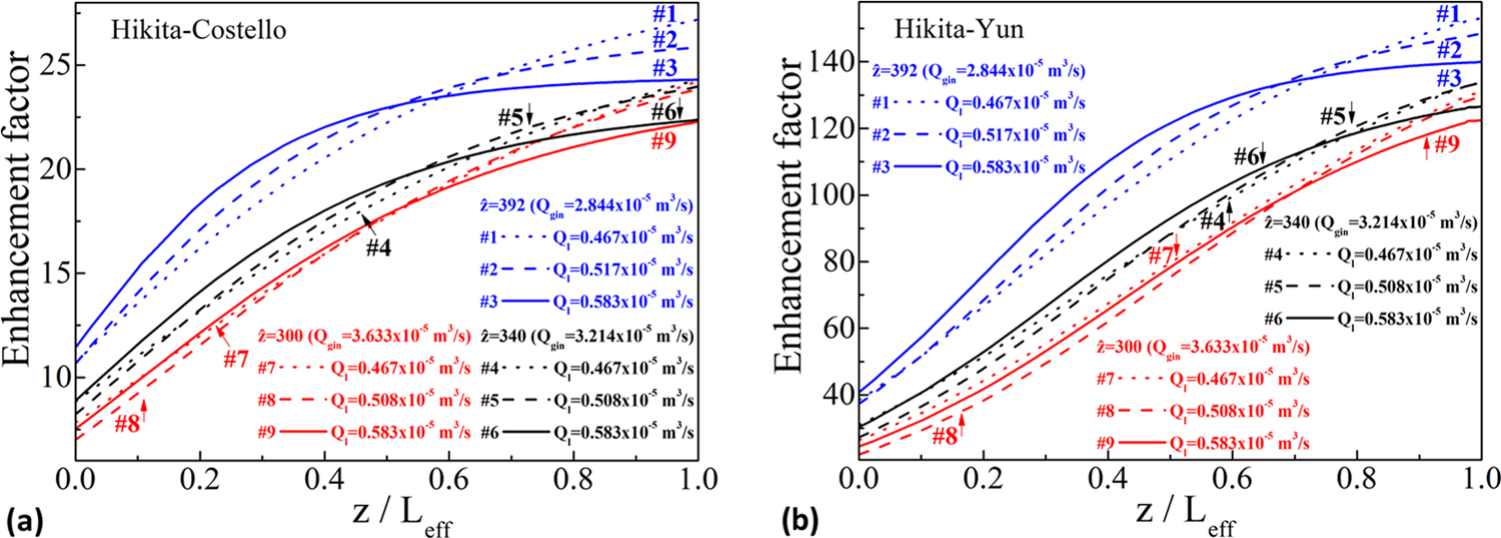
Variation of the enhancement factor along the shell side for all experimental sets for the Hikita–Costello (a) and the Hikita–Yun (b) pairs.

Figure 8 illustrates the variation of the enhancement factors along the shell-side axial distance for the two shell-side mass transfer correlations. First, since the shell-side mass transfer coefficient for the Costello et al. correlation is higher than that of the Yun et al. value, the enhancement factor values are higher for the Hikita–Yun pair than those for the Hikita–Costello pair due to eq 25—the “enhancement” compared to the physical-absorption case.

When less CO_2_ flows in the lumen (increasing *ẑ*–decreasing *Q*_g,in_) to react with the same amount of liquid (same line-style), the remaining *free* CO_2_ (not having reacted) on the shell side is less and the remaining DEA concentration is higher than that in the case with a higher initial gas flow rate on the lumen side. Thus, the infinite enhancement factor, *E*_∞_, is higher due to eq 26 and the Hatta number, *Ha*, is higher due to eq 25, which lead to higher enhancement factors, E, on the shell side due to eq 24.

In Section 4.2, it is indicated that inclusion of an enhancement in the liquid-filled part of the membrane equations yields higher wetting values. The higher enhancement factor values for smaller feed flow rates (higher *ẑ*, Figure 8) might indicate the missing “calibrating” parameter in the liquid-filled part of the membrane equations. Consider the Hikita–Costello pair in Figure 4 and assume for one that the “deviation” of wetting from a single value for the same liquid flow rate may be attributed to the absence of reaction enhancement within the liquid-filled part of the pores. The wetting values must then increase to “match” with those of the less *ẑ*-value-sets (as implied by Figure 4), which can be done by incorporating a comparably higher enhancement value for the higher *ẑ*-value-sets. Nevertheless, there would still be the need to define a reference value of the enhancement in the liquid-filled pores for a set, or to know beforehand the value of, say, the apparent wetting, *x*′/*E*′, which would be the same for all *ẑ* for the same liquid flow rate. However, the authors of the current study cannot provide any theoretical or empirical justification on the basis of this deduction, and the Hikita–Yun pair results do not imply any relation between the wetting and the gas flow rates.

**Figure 9:**
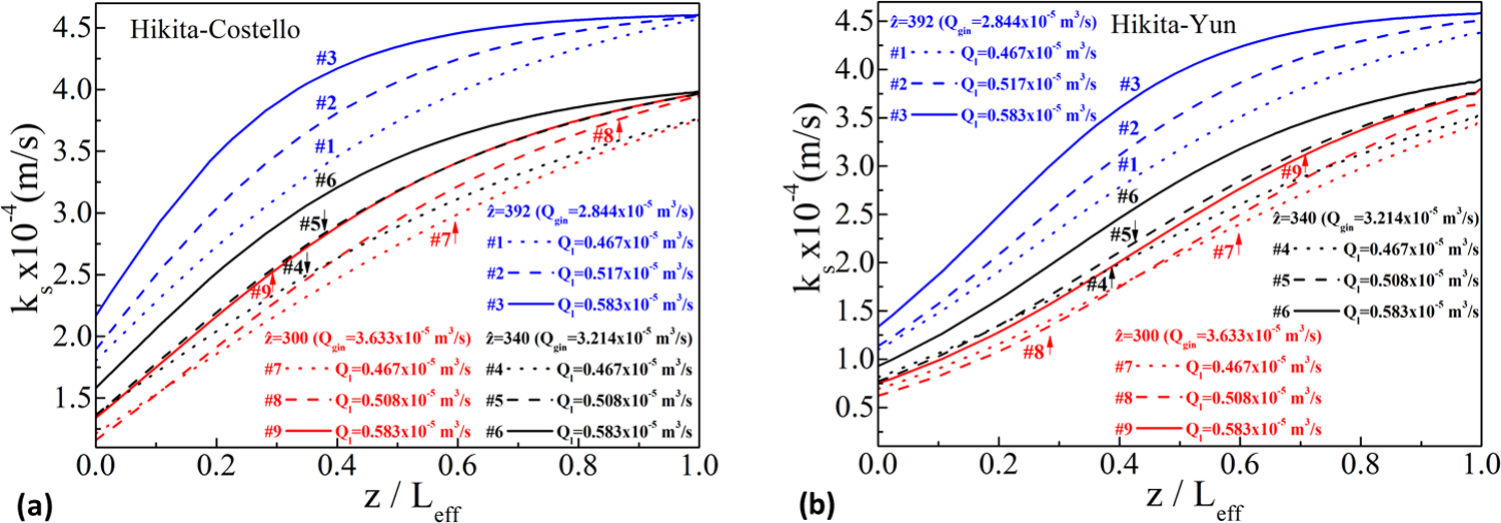
Variation of the apparent shell-side mass transfer coefficient, *k*_s_, along dimensionless z/L_eff_ for all experimental sets for the Hikita–Costello (a) and the Hikita–Yun (b) pairs.

Figure 9 presents the profiles of the apparent shell-side mass transfer coefficient values along z/L_eff_ for all experimental sets as derived with the use of either Hikita–Costello or Hikita–Yun pairs. First, it may be noticed that they follow quite the same trend as the enhancement factors; they exhibit higher values for higher liquid flow rates due to the increase of the *k*_*s*, *o*_ values. Second, the values when using the two correlations are comparable, but not identical, in the whole length of the shell side.

**Figure 10:**
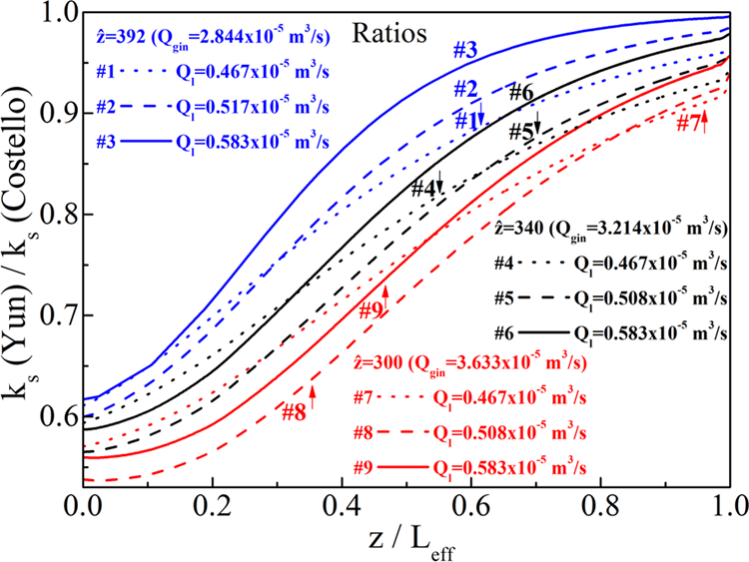
Ratio of the apparent shell-side mass transfer coefficient values (Yun:Costello) along the shell side for all experimental sets.

Figure 10 depicts the ratio of the apparent shell-side mass transfer coefficient values (values derived from the Yun correlation divided by those by Costello) along the shell side for all experimental sets. It is seen that they cannot be assumed equal along the length of the shell. If they were identical (or close to each other) in the whole domain, the apparent mass transfer coefficient, *k*_s_, would be independent of *k*_s,o_, regardless of the shell-side correlation chosen. In that case, for each experimental set, the effective membrane mass transfer coefficient, *k*_m,eff_, should have been the same, yielding the same wetting value for all shell-side correlations; also (and equivalently), the enhancement factor value should have been equal to the Hatta number (see eq 27), but this is not the case as explained below.

**Figure 11:**
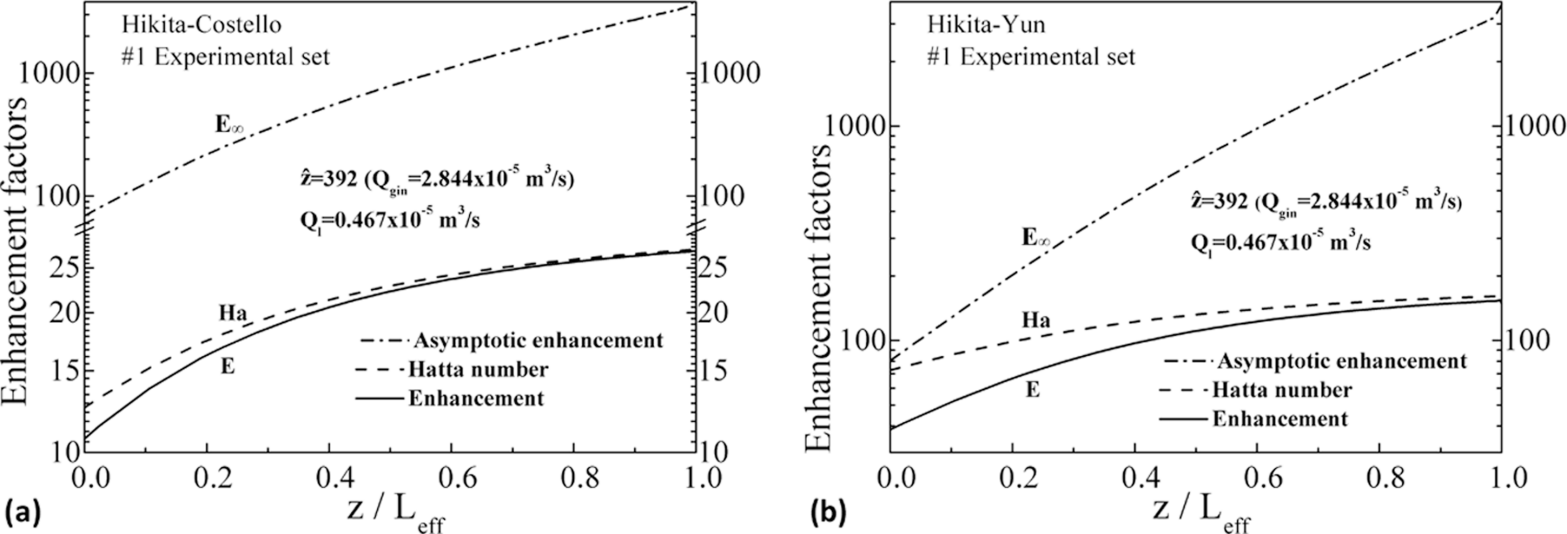
Variation of *E*, *E*_∞_, and Ha along the shell side for the No. 1 experimental set for the Hikita–Costello (a) and the Hikita–Yun (b) pairs.

The variation of the asymptotic enhancement factor, *E*_∞_, the Hatta number, *Ha*, and the enhancement factor, *E*, for the No. 1 experimental set is shown in Figure 11 for the two shell-side correlations and also for all experimental sets. For large values of *E*_∞_ > 1000 and 10 < *Ha* < 100, the enhancement factor, *E*, is almost equal to the *Ha* number, as indicated in the general graph of Figure 2. For smaller *E*_∞_ and *Ha*, or for values of *Ha* > 100 regardless of the value of *E*_∞_, *E* deviates from *Ha* (more evident in the Hikita–Yun pair). Demonstratively, in Figure 10, the apparent shell-side mass transfer coefficient values of the two correlations are closer to each other at the upper right z-domain, where the values of *Ha* and *E* are nearly equal. The results indicate that it is incorrect to consider equal the values of the Hatta number and the enhancement factor in the whole shell domain so that the choice of the shell-side correlations has an appreciable impact on the overall analysis, especially for the determination of the wetting values.

The variation of the variables of interest would have been less pronounced for higher concentrations of DEA in the aqueous solution, that is, by working in a mass transfer regime not dependent on the process liquid flow conditions, an assumption that can be tested by comparing the values of the Hatta number and the enhancement factor. In that case, the profiles on the shell side, such as those of *k*_s_ and *K*_ext_, would become smoother (for each experiment), and the “real” computational wetting would “converge” to a single value regardless of the choice of the shell-side mass transfer correlation. This might be a guiding principle even in scaled-up membrane modules when considering a multicomponent mixture (e.g., biogas consisting of CH_4_, CO_2_, and H_2_S) with at least two components taking part in the reaction (e.g., CO_2_ and H_2_S with DEA); the wetting could then be found by matching, e.g., the CO_2_ experimental-simulation results, and the H_2_S concentration could be inferred from this procedure and compared with the experimental results. At the same time, the validity of the overall model and assumptions is assessed.

**Figure 12:**
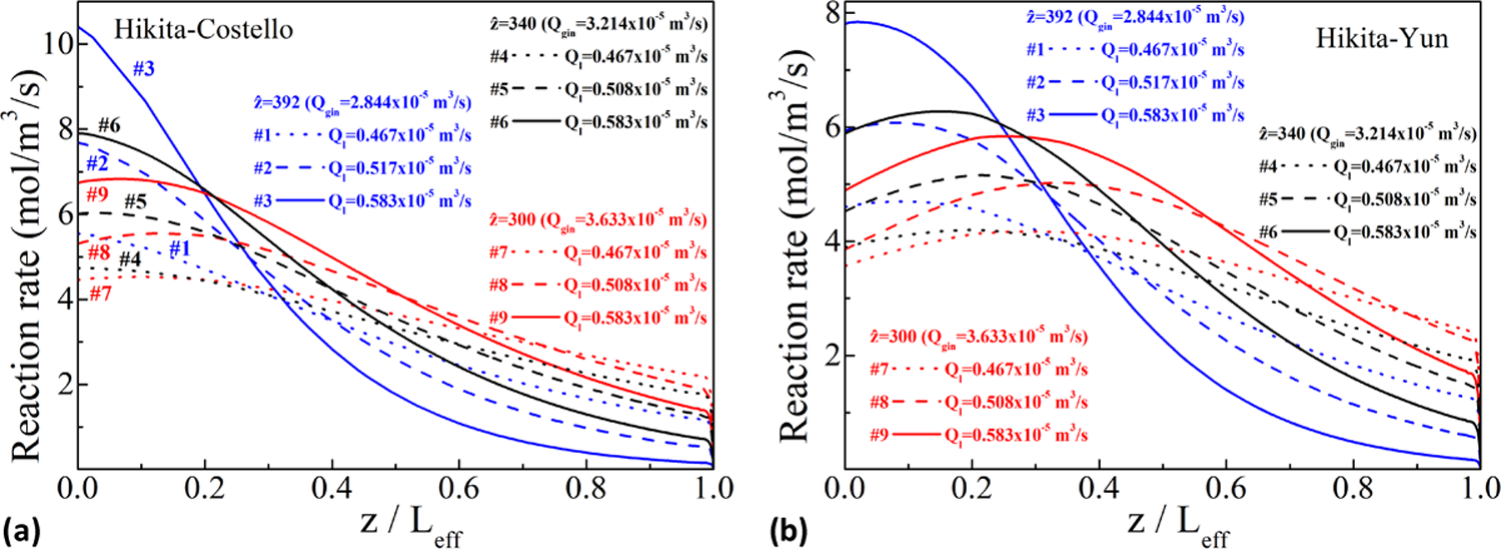
Variation of the reaction rate along the shell side for all experimental sets for the Hikita–Costello (a) and the Hikita–Yun (b) pairs.

The reaction rate profiles illustrated in Figure 12 show that they are higher in the beginning (near *z* = 0) since a higher amount of CO_2_ enters the shell-side zone near the inlet, while DEA is still present in abundance. This is more evident for higher liquid and smaller gas flow rates (e.g., experiment #3), while for smaller liquid and higher gas flow rates (e.g., experiment #7), the reaction rate obtains comparable values in the whole shell side. The smallest amount of CO_2_ near the exit of the module is the reason for the greatest *E*_∞_ values in this region due to eq 26; for these *E*_∞_ values, the enhancement factor, *E*, is almost equal to the Hatta number, *Ha*, as also indicated in the previous paragraphs.

**Figure 13:**
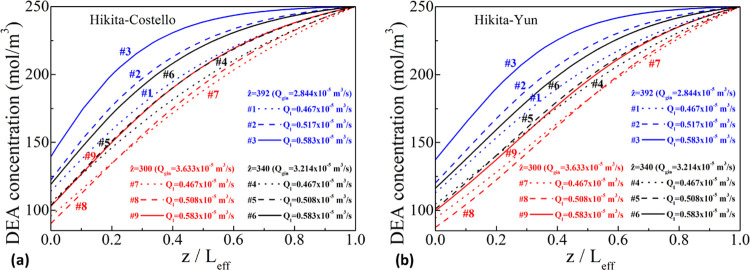
Variation of DEA concentration along the shell side for all experimental sets for the Hikita–Costello (a) and the Hikita–Yun (b) pairs.

**Figure 14:**
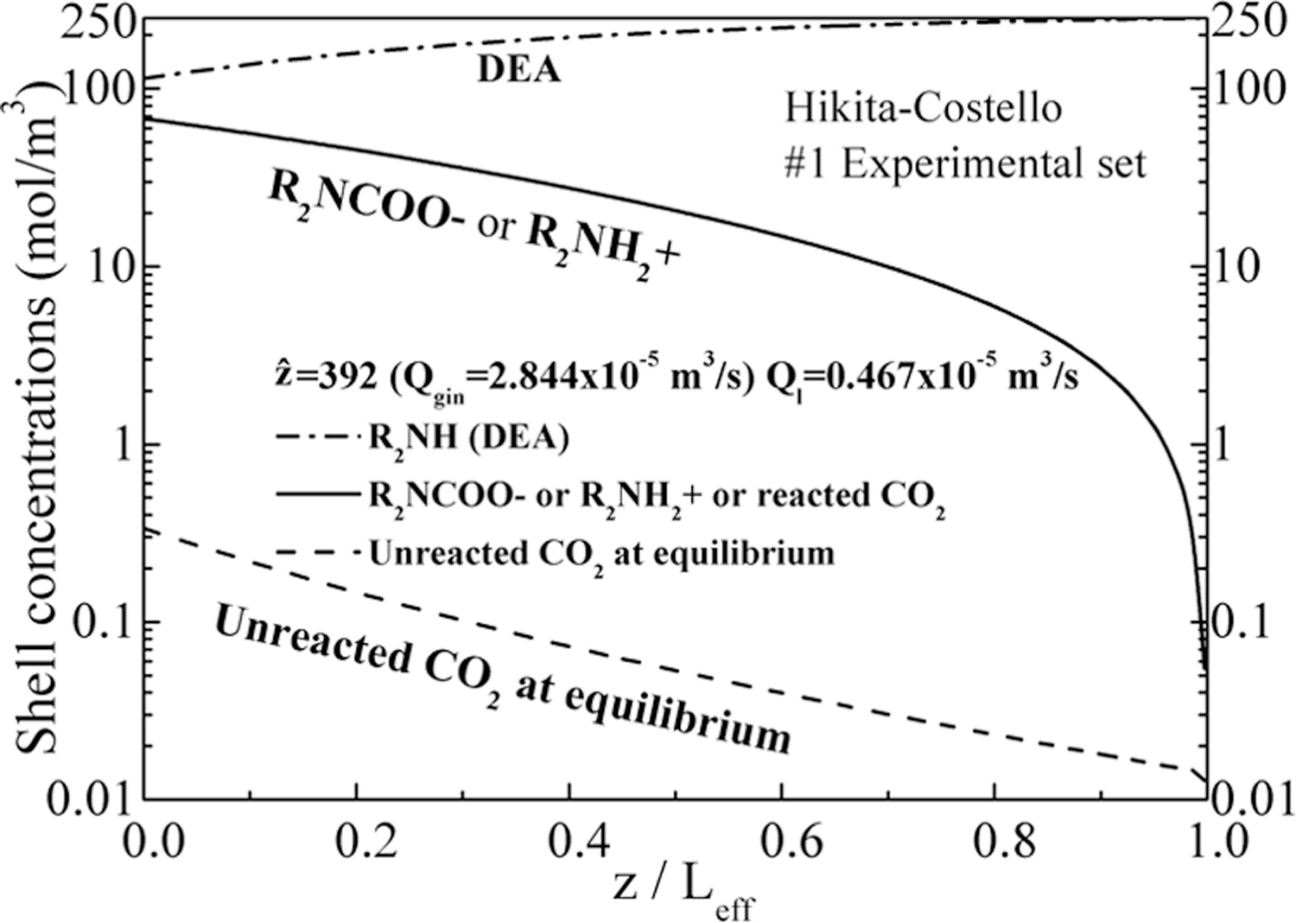
Variation of ions, DEA, and CO_2_ at equilibrium along the shell side for the No. 1 experimental set for the Hikita–Costello pair.

The DEA concentration variation for either correlation is indistinguishable, as shown in Figure 13. The aqueous solution of DEA enters counter-currently (at *z* = *L*_eff_) with an initial concentration of 250 mol/m^3^ (0.25 M), and its concentration diminishes when flowing toward *z* = 0. The smallest final DEA concentration (at *z* = 0) (i.e., the highest DEA consumption) is found for the highest biogas feed flow rate, i.e., for the #7 and #8 experimental sets. Figure 14 illustrates all of the species variation on the shell side for the #1 experimental set. The ion species concentrations (CH_2_CH_2_OH)_2_NH_2_^+^ (diethanol ammonium) and (CH_2_CH_2_OH)_2_NCOO- (carbamate of diethanolamine), and consequently the reacted CO_2_ according to the stoichiometry of the reaction, increase equally along *z* (from the right to the left due to the countercurrent mode of operation). There is also a small quantity of free (unreacted) CO_2_ at equilibrium with the solvent.

## 5 Conclusions

In this study, the basic principles and assumptions of a steady-state mass transfer model are assessed with the aid of a series of experiments in a gas–liquid contact membrane module concerning biogas upgrade using an aqueous solution of DEA (0.25 M). Experimental data show that, at ambient conditions, CO_2_ removal ranges between 67 and 99% combined with the highest possible recovery of CH_4_ with biogas flow rates in the range of 2.8 × 10^–5^–3.6 × 10^–5^ m^3^/s and solvent flow rates within 0.47 × 10^–5^–0.58 × 10^–5^ m^3^/s. For the experimental data set, a correlation has been developed interpolating CO_2_ removal with respect to dimensionless numbers (in effect, depending on the gas and liquid flow rates), most convenient for design purposes.

The main postulations of the model are that the binary biogas mixture variation can be adequately described in a 2-D representation in fibers with a fully developed parabolic velocity profile adjusted to the variation of the gas flow rate in the fibers due to pressure drop and loss of CO_2_, while a third-order reaction term for CO_2_-DEA consumption can adequately predict the 1-D shell-side variation. The diffusing component has to sequentially overcome mass transfer resistance in the gas-filled part of the membrane pores, followed by the resistance of the remaining liquid-filled membrane pores imposed by any liquid intrusion, before diffusing into the liquid film and reacting with the solvent. The wetting value is then calculated by matching the experimental results and the computational predictions. A varying-with-length combined mass transfer coefficient is a key element in the mass transfer analysis, which provides a broad and rather unrestrained description of the overall model and the shell computational compartment. Two literature shell-side mass transfer correlations alluding to physical absorption are qualified for the simulation runs in order to quantify the enhancement due to reaction.

The main findings of the study are that the wetting values calculated are concentrated close to each other for the same liquid flow rate without considerably depending on the gas flow rate, especially when applying the Hikita–Yun (reaction rate–shell-side correlation) pair. Furthermore, the calculated wetting diminishes with increasing liquid flow rate, a result that is consistent with previous modeling attempts of the authors of the current study and relevant literature indications. The assumption of enhanced mass transfer in the liquid-filled part of the membrane pores due to reaction is examined, revealing a substantial decrease in the corresponding mass transfer resistance and objectionable computational wetting values, certainly not in accordance with literature findings. To the best of the authors’ knowledge, there is not yet a unified theoretical approach to predict (any) enhancement factor in the liquid-filled part of the membrane pores.

The mass transfer regime for a DEA concentration equal to 0.25 M is not exactly close to the flow-independent behavior since the Hatta numbers and the enhancement factors are not equal; thus, the choice of the shell-side correlation has an appreciable impact on the overall analysis, especially for the determination of the wetting values.

Although the analysis and the calculated values presented in this study indicate that the wetting evaluation is still an elusive task that cannot be isolated from the shell-side mass transfer coefficient in the gas–liquid membrane modeling studies, it is suggested that the very concept of the film model, the resistance-in-series model, and the associated connotations are fundamentally consistent with the experimental results and the notion of the partial wetting of membrane pores.

Based on the aforementioned findings, targeted experimental campaigns will be conducted to examine modeling scenarios of multicomponent mixtures and altered biogas composition, alleviation of the assumptions of isothermal operation or a uniform membrane porous network, increased DEA concentrations and cross-flow modules, and other operational parameters covering a set of more realistic working scenarios.

## Supporting Information Available

Appendix A, Additional equations for the membrane-based gas absorption system; Appendix B, Physicochemical properties; Appendix C, Additional literature review, figures, and comments (PDF)

## Nomenclature

*a*_*v*_: specific fiber-to-shell surface area (m^–1^), see the Supporting Information file
*C*_*i*_: concentration of the diffusing component in the lumen (mol/m^3^), *i* = CO_2_, CH_4_
*C*_*i*,o_: feed concentration of component *i* in the lumen (mol/m^3^)
*C*_s,*i*,*z*_: concentration of component *i* in the solvent (mol/m^3^), *i* = CO_2_, CH_4_, DEA
CO_2_ Removal: CO_2_ removal efficiency of the absorption process (%), see the Supporting Information file
*D*_eff, *i*_: membrane effective gas diffusion coefficient of component *i* (m^2^/s)
*d*_h_: shell-side hydraulic diameter (m), see the Supporting Information file
*D*_*i*_: gas–gas diffusion coefficient of the diffusing component *i* in the lumen (m^2^/s)
*D*_*i*,amine_: solute diffusion coefficient into the solvent of component *i* (m^2^/s), see the Supporting Information file
*E*,*E*_avg_,*E*_∞_: enhancement, average enhancement, and asymptotic enhancement factors, respectively
*Ha*: Hatta number
*Gz*: Graetz number
*H*_A, solv_: Henry’s constant of the dissolved gas (Pa·m^3^/mol), see the Supporting Information file
*K*_ext_: combined (shell and membrane) or external mass transfer coefficient (m/s)
*k*_mg_: gas-filled membrane mass transfer coefficient, inverse *R*_mg_ (m/s)
*k*_ml_: liquid-filled membrane mass transfer coefficient, inverse *R*_ml_ (m/s)
*k*_R_: reaction rate constant (L^2^/mol^2^/s)
*k*_s_: apparent shell-side mass transfer coefficient (m/s)
*k*_s,o_: not enhanced shell-side mass transfer coefficient (alluding to physical absorption) (m/s)
*L*_eff_: effective fiber length (m), see Table 1
*m*: dimensionless equilibrium coefficient (Henry’s constant)
*m**: adjusted gas-to-liquid flow rate ratio accounting for the equilibrium coefficient (*mQ*_*g*, *in*_/*Q*_*l*_)
*n*_*i*_: species stoichiometric coefficient for the shell-side reaction
*N*_f_: number of fibers, see Table 1
*Q*_g,in_, *Q*_g,out_: inlet and outlet volumetric flow rates in the lumen (m^3^/s), respectivey; see the Supporting Information file
*Q*_l_: solvent volumetric flow rate on the shell side (m^3^/s)
*r*: radial distance across the fiber (m)
*R*: gas constant (Pa·m^3^/mol/K)
Rate: shell-side reaction rate (mol/m^3^/s or mol/L/s in its definition)
*Re*_s_: shell-side Reynolds number
*R*_f_: fiber radius (m), see Table 1
*R*_m_: total membrane mass transfer resistance (s/m)
*R*_mg_: gas-filled membrane mass transfer resistance not accounting for wetting (s/m)
*R*_ml_: liquid-filled membrane mass transfer resistance not accounting for wetting (s/m)
*R*_mg, eff_: effective gas-filled membrane mass transfer resistance accounting for wetting (s/m)
*R*_ml,eff_: effective liquid-filled membrane mass transfer resistance accounting for wetting (s/m)
*R*_s_: apparent shell-side mass transfer resistance (s/m)
*Sc*_s_: shell-side Schmidt number
*Sh*_s_: shell-side Sherwood number
*T*: temperature (K)
*u*: lumen average velocity (m/s)
*u*_int_: shell-side interstitial (within the voids) velocity (m/s), see the Supporting Information file
*x*: membrane wetting
*y*_out,exp_: experimental CO_2_ molar fraction at the exit of the lumen side, see Table 2
*y*_out,sim_: model prediction of CO_2_ molar fraction at the exit of the lumen side, mixed-cup quantity
*z*: axial distance (m)
*ẑ*: inverse Gz number
ε_f_: membrane porosity, see Table 1
η_DEA,H_2_O_: kinematic viscosity of aqueous solution of DEA (m^2^/s), see the Supporting Information file
τ: membrane tortuosity, see Table 1
φ: packing fraction, see Table 1

## References

[ref1] Bauer F.; Persson T.; Hulteberg C.; Tamm D. Biogas upgrading – Technology overview, comparison and perspectives for the future. Biofuel. Bioprod. Bioref. 2013, 7, 499–511. 10.1002/bbb.1423.

[ref2] Khan M. U.; Lee J. T. E.; Bashir M. A.; Dissanayake P. D.; Ok Y. S.; Tong Y. W.; Shariati M. A.; Wu S.; Ahring B. K. Current status of biogas upgrading for direct biomethane use: A review. Renewable Sustainable Energy Rev. 2021, 149, 111343 10.1016/j.rser.2021.111343.

[ref3] European Biogas Association (EBA) Statistical Report2022 (webinar) https://www.europeanbiogas.eu/__trashed-3/ (accessed January 2024).

[ref4] European Commission Commission Staff Working Document, SWD (2022) 230 final; COM(2023) 796 final, 2022.

[ref5] Pantoleontos G.; Kaldis S. P.; Koutsonikolas D.; Grammelis P.; Sakellaropoulos G. P. CO_2_ absorption in a mini-module membrane contactor. In Global Warming; Springer Science+Business Media, LLC, 2010; pp 307–313.

[ref6] Pantoleontos G.; Kaldis S. P.; Koutsonikolas D.; Skodras G.; Sakellaropoulos G. P. Analytical and numerical solutions of the mass continuity equation in the lumen side of a hollow-fiber membrane contactor with linear or nonlinear boundary conditions. Chem. Eng. Commun. 2010, 197, 709–732. 10.1080/00986440903288039.

[ref7] Koutsonikolas D.; Pantoleontos G.; Mavroudi M.; Kaldis S.; Pagana A.; Kikkinides E. S.; Konstantinidis D. Pilot tests of CO_2_ capture in brick production industry using gas–liquid contact membranes. Int. J. Energy Environ. Eng. 2016, 7, 61–68. 10.1007/s40095-015-0193-x.

[ref8] Pantoleontos G.; Theodoridis T.; Mavroudi M.; Kikkinides E. S.; Koutsonikolas D.; Kaldis S. P.; Pagana A. E. Modelling, simulation, and membrane wetting estimation in gas-liquid contacting processes. Can. J. Chem. Eng. 2017, 95, 1352–1363. 10.1002/cjce.22790.

[ref9] Pantoleontos G.; Anagnostara I. M.; Syrigou M.; Konstandopoulos A. G. Solutions of the mass continuity equation in hollow fibers for fully developed flow with some notes on the Lévêque correlation. Carbon Capt. Sci. Technol. 2022, 2, 100027 10.1016/j.ccst.2021.100027.

[ref10] Crespo J. G.; Coelhoso I. M.; Viegas M. C. Membrane Contactors: Membrane Separations. In Encyclopedia of Separation Science; Academic Press, 2007; pp 3303–3311.

[ref11] Mansourizadeh A.; Ismail A. F. Hollow fiber gas–liquid membrane contactors for acid gas capture: A review. J. Hazard. Mater. 2009, 171, 38–53. 10.1016/j.jhazmat.2009.06.026. 19616376

[ref12] Kerber J.; Repke J.-U. Mass transfer and selectivity analysis of a dense membrane contactor for upgrading biogas. J. Membr. Sci. 2016, 520, 450–464. 10.1016/j.memsci.2016.08.008.

[ref13] Mavroudi M.; Kaldis S. P.; Sakellaropoulos G. P. A study of mass transfer resistance in membrane gas–liquid contacting processes. J. Membr. Sci. 2006, 272, 103–115. 10.1016/j.memsci.2005.07.025.

[ref14] Ibrahim M. H.; El-Naas M. H.; Zhang Z.; Van der Bruggen B. CO_2_ capture using hollow fiber membranes: a review of membrane wetting. Energy Fuel 2018, 32, 963–978. 10.1021/acs.energyfuels.7b03493.

[ref15] Malek A.; Li K.; Teo W. K. Modeling of microporous hollow fiber membrane modules operated under partially wetted conditions. Ind. Eng. Chem. Res. 1997, 36, 784–793. 10.1021/ie960529y.

[ref16] Rangwala H. A. Absorption of carbon dioxide into aqueous solutions using hollow fiber membrane contactors. J. Membr. Sci. 1996, 112, 229–240. 10.1016/0376-7388(95)00293-6.

[ref17] Mavroudi M.; Kaldis S. P.; Sakellaropoulos G. P. Reduction of CO_2_ emissions by a membrane contacting process. Fuel 2003, 82, 2153–2159. 10.1016/S0016-2361(03)00154-6.

[ref18] Zhang H.-Y.; Wang R.; Liang D. T.; Tay J. H. Theoretical and experimental studies of membrane wetting in the membrane gas-liquid contacting process for CO_2_ absorption. J. Membr. Sci. 2008, 308, 162–170. 10.1016/j.memsci.2007.09.050.

[ref19] Lu J.-G.; Zheng Y.-F.; Cheng M.-D.; Wang L.-J. Wetting mechanism in mass transfer process of hydrophobic membrane gas absorption. J. Membr. Sci. 2008, 308, 180–190. 10.1016/j.memsci.2007.09.051.

[ref20] Mansourizadeh A.; Ismail A. F.; Matsuura T. Effect of operating conditions on the physical and chemical CO_2_ absorption through the PVDF hollow fiber membrane contactor. J. Membr. Sci. 2010, 353, 192–200. 10.1016/j.memsci.2010.02.054.

[ref21] Cui L.; Ding Z.; Liu L.; Li Y. Modelling and experimental study of membrane wetting in microporous hollow fiber membrane contactors. Can. J. Chem. Eng. 2015, 93, 1254–1265. 10.1002/cjce.22210.

[ref22] Yu H.; Thé J.; Tan Z.; Feng X. Modeling SO_2_ absorption into water accompanied with reversible reaction in a hollow fiber membrane contactor. Chem. Eng. Sci. 2016, 156, 136–146. 10.1016/j.ces.2016.09.020.

[ref23] Fougerit V.; Pozzobon V.; Pareau D.; Théoleyre M.-A.; Stambouli M. Gas-liquid absorption in industrial cross-flow membrane contactors: Experimental and numerical investigation of the influence of transmembrane pressure on partial wetting. Chem. Eng. Sci. 2017, 170, 561–573. 10.1016/j.ces.2017.03.042.

[ref24] Evren V. A numerical approach to the determination of mass transfer performances through partially wetted microporous membranes: transfer of oxygen to water. J. Membr. Sci. 2000, 175, 97–110. 10.1016/S0376-7388(00)00401-4.

[ref25] Porcheron F.; Drozdz S. Hollow fiber membrane contactor transient experiments for the characterization of gas/liquid thermodynamics and mass transfer properties. Chem. Eng. Sci. 2009, 64, 265–275. 10.1016/j.ces.2008.09.035.

[ref26] El-Naas M. H.; Al-Marzouqi M.; Marzouk S. A.; Abdullatif N. Evaluation of the removal of CO_2_ using membrane contactors: membrane wettability. J. Membr. Sci. 2010, 350, 410–416. 10.1016/j.memsci.2010.01.018.

[ref27] Boributh S.; Assabumrungrat S.; Laosiripojana N.; Jiraratananon R. Effect of membrane module arrangement of gas–liquid membrane contacting process on CO_2_ absorption performance: A modeling study. J. Membr. Sci. 2011, 372, 75–86. 10.1016/j.memsci.2011.01.034.

[ref28] Boributh S.; Rongwong W.; Assabumrungrat S.; Laosiripojana N.; Jiraratananon R. Mathematical modeling and cascade design of hollow fiber membrane contactor for CO_2_ absorption by monoethanolamine. J. Membr. Sci. 2012, 401–402, 175–189. 10.1016/j.memsci.2012.01.048.

[ref29] Wang Z.; Fang M.; Yan S.; Yu H.; Wei C.-C.; Luo Z. Optimization of blended amines for CO_2_ absorption in a hollow-fiber membrane contactor. Ind. Eng. Chem. Res. 2013, 52, 12170–12182. 10.1021/ie401676t.

[ref30] Rongwong W.; Fan C.; Liang Z.; Rui Z.; Idem R. O.; Tontiwachwuthikul P. Investigation of the effects of operating parameters on the local mass transfer coefficient and membrane wetting in a membrane gas absorption process. J. Membr. Sci. 2015, 490, 236–246. 10.1016/j.memsci.2015.04.071.

[ref31] Kreulen H.; Smolders C. A.; Versteeg G. F.; van Swaaij W. P. M. Microporous hollow fibre membrane modules as gas-liquid contactors. Part 1. Physical mass transfer processes. A specific application: Mass transfer in highly viscous liquids. J. Membr. Sci. 1993, 78, 197–216. 10.1016/0376-7388(93)80001-E.

[ref32] Drew T. B.; Hogan J. J.; McAdams W. H. Heat transfer in stream-line flow. Ind. Eng. Chem. 1931, 23, 936–945. 10.1021/ie50260a020.

[ref33] Newman J. Extension of the Lévêque Solution; University of California, Ernest O. Lawrence Radiation Laboratory, June 1967.

[ref34] Yin Y.; Cao Z.; Gao H.; Sema T.; Na Y.; Xiao M.; Liang Z.; Tontiwachwuthikul P. Experimental measurement and modeling prediction of mass transfer in a hollow fiber membrane contactor using tertiary amine solutions for CO_2_ absorption. Ind. Eng. Chem. Res. 2022, 61, 9632–9643. 10.1021/acs.iecr.2c00637.

[ref35] Qin Y.; Cabral J. M. S. Lumen mass transfer in hollow-fiber membrane processes with constant external resistances. AIChE J. 1997, 43, 1975–1988. 10.1002/aic.690430807.

[ref36] Asimakopoulou A.; Koutsonikolas D.; Kastrinaki G.; Skevis G. Innovative gas-liquid membrane contactor systems for carbon capture and mineralization in energy intensive industries. Membranes 2021, 11, 271 10.3390/membranes11040271. 33917973 PMC8068349

[ref37] Vogler S.; Braasch A.; Buse G.; Hempel S.; Schneider J.; Ulbricht M. Biogas conditioning using hollow fiber membrane contactors. Chem. Ing. Technol. 2013, 85, 1254–1258. 10.1002/cite.201200235.

[ref38] 3M Liqui-Cel Membrane Contactors: https://multimedia.3m.com/mws/media/1412485O/3m-liqui-cel-membrane-contactors-liquid-degasgaslc-1096-pdf.pdf & https://www.3m.com/3M/en_US/p/dc/v000425586; 3M Liqui-Cel MM-1.7 × 5.5 Series Membrane Contactor: https://multimedia.3m.com/mws/media/1412492O/3m-liqui-cel-mm-1-7x5-5-series-membrane-contactorlc-1007.pdf; Liqui-cel Membrane Contactors. Design and Operating Procedures: https://gmpua.com/Equipment/Deaeration/Operating%20Procedure.pdf.

[ref39] Delgado J. A.; Uguina M. A.; Sotelo J. L.; Águeda V. I.; Sanz A. Simulation of CO_2_ absorption into aqueous DEA using a hollow fiber membrane contactor: Evaluation of contactor performance. Chem. Eng. J. 2009, 152, 396–405. 10.1016/j.cej.2009.04.064.

[ref40] Lee A. S.; Eslick J. C.; Miller D. C.; Kitchin J. R. Comparisons of amine solvents for post-combustion CO_2_ capture: A multi-objective analysis approach. Int. J. Greenh. Gas Con. 2013, 18, 68–74. 10.1016/j.ijggc.2013.06.020.

[ref41] Yeon S.-H.; Lee K.-S.; Sea B.; Park Y.-I.; Lee K.-H. Application of pilot-scale membrane contactor hybrid system for removal of carbon dioxide from flue gas. J. Membr. Sci. 2005, 257, 156–160. 10.1016/j.memsci.2004.08.037.

[ref42] Xu Y.; Malde C.; Wang R. Correlating physicochemical properties of commercial membranes with CO_2_ absorption performance in gas-liquid membrane contactor. J. Membrane Sci. Res. 2006, 6, 30–39.

[ref43] deMontigny D.; Tontiwachwuthikul P.; Chakma A. Using polypropylene and polytetrafluoroethylene membranes in a membrane contactor for CO_2_ absorption. J. Membr. Sci. 2006, 277, 99–107. 10.1016/j.memsci.2005.10.024.

[ref44] Lu J.-G.; Zheng Y.-F.; Cheng M.-D.; Wang L.-J. Effects of activators on mass-transfer enhancement in a hollow fiber contactor using activated alkanolamine solutions. J. Membr. Sci. 2007, 289, 138–149. 10.1016/j.memsci.2006.11.042.

[ref45] Versteeg G. F.; van Swaaij W. P. M. Solubility and diffusivity of acid gases (carbon dioxide, nitrous oxide) in aqueous alkanolamine solutions. J. Chem. Eng. Data 1988, 33, 29–34. 10.1021/je00051a011.

[ref46] Michalis V. K.; Moultos O. A.; Tsimpanogiannis I. N.; Economou I. G. Molecular dynamics simulations of the diffusion coefficients of light n-alkanes in water over a wide range of temperature and pressure. Fluid Phase Equilib. 2016, 407, 236–242. 10.1016/j.fluid.2015.05.050.

[ref47] Moultos O. A.; Tsimpanogiannis I. N.; Panagiotopoulos A. Z.; Economou I. G. Self-diffusion coefficients of the binary (H_2_O + CO_2_) mixture at high temperatures and pressures. J. Chem. Thermodyn. 2016, 93, 424–429. 10.1016/j.jct.2015.04.007.

[ref48] Polat H. M.; Coelho F. M.; Vlugt T. J. H.; Franco L. F. M.; Tsimpanogiannis I. N.; Moultos O. A. Diffusivity of CO_2_ in H_2_O: a review of experimental studies and molecular simulations in the bulk and in confinement J. Chem. Eng. Data 2024, 10.1021/acs.jced.3c00778. PMC11480918 39417156

[ref49] Tsimpanogiannis I. N.; Maity S.; Celebi A. T.; Moultos O. A. An engineering model for predicting the intradiffusion coefficients of hydrogen and oxygen in vapor, liquid and supercritical water based on molecular dynamics simulations. J. Chem. Eng. Data 2011, 66, 3226–3244. 10.1021/acs.jced.1c00300.

[ref50] Kumar P. S.; Hogendoorn J. A.; Feron P. H. M.; Versteeg G. F. New absorption liquids for the removal of CO_2_ from dilute gas streams using membrane contactors. Chem. Eng. Sci. 2002, 57, 1639–1651. 10.1016/S0009-2509(02)00041-6.

[ref51] Danckwerts P. V. Gas-Liquid Reactions; McGraw-Hill, 1970.

[ref52] Yang M.-C.; Cussler E. L. Designing hollow-fiber contactors. AIChE J. 1986, 32, 1910–1916. 10.1002/aic.690321117.

[ref53] Prasad R.; Sirkar K. K. Dispersion-free solvent extraction with microporous hollow-fiber modules. AIChE J. 1988, 34, 177–188. 10.1002/aic.690340202.

[ref54] Kreulen H.; Versteeg G. F.; Smolders C. A.; van Swaaij W. P. M. Selective removal of H_2_S from sour gas with microporous membranes. Part I. Application in a gas-liquid system. J. Membr. Sci. 1992, 73, 293–304. 10.1016/0376-7388(92)80136-8.

[ref55] Scholes C. A.; Simioni M.; Qader A.; Stevens G. W.; Kentish S. E. Membrane gas–solvent contactor trials of CO_2_ absorption from syngas. Chem. Eng. J. 2012, 195–196, 188–197. 10.1016/j.cej.2012.04.034.

[ref56] Estay H.; Troncoso E.; Ruby-Figueroa R.; Romero J. Performance evaluation of mass transfer correlations in the GFMA process: A review with perspectives to the design. J. Membr. Sci. 2018, 554, 140–155. 10.1016/j.memsci.2018.02.064.

[ref57] Basu R.; Prasad R.; Sirkar K. K. Nondispersive membrane solvent back extraction of phenol. AIChE J. 1990, 36, 450–460. 10.1002/aic.690360314.

[ref58] Yun C. H.; Prasad R.; Guha A. K.; Sirkar K. K. Hollow fiber solvent extraction removal of toxic heavy metals from aqueous waste streams. Ind. Eng. Chem. Res. 1993, 32, 1186–1195. 10.1021/ie00018a026.

[ref59] Costello M. J.; Fane A. G.; Hogan P. A.; Schofield R. W. The effect of shell side hydrodynamics on the performance of axial flow hollow fibre modules. J. Membr. Sci. 1993, 80, 1–11. 10.1016/0376-7388(93)85127-I.

[ref60] Versteeg G. F.; van Swaaij W. P. M. On the kinetics between CO_2_ and alkanolamines both in aqueous and non-aqueous solutions—I. Primary and secondary amines. Chem. Eng. Sci. 1988, 43, 573–585. 10.1016/0009-2509(88)87017-9.

[ref61] Versteeg G. F.; Oyevaar M. H. The reaction between CO_2_ and diethanolamine at 298 K. Chem. Eng. Sci. 1989, 44, 1264–1268. 10.1016/0009-2509(89)87026-5.

[ref62] Hikita H.; Asai S.; Ishikawa H.; Honda M. The kinetics of reactions of carbon dioxide with monoethanolamine, diethanolamine and triethanolamine by a rapid mixing method. Chem. Eng. J. 1977, 13, 7–12. 10.1016/0300-9467(77)80002-6.

[ref63] Onda K.; Sada E.; Kobayashi T.; Fujine M. Gas absorption accompanied by complex chemical reactions – Reversible chemical reactions. Chem. Eng. Sci. 1970, 25, 753–760. 10.1016/0009-2509(70)85110-7.

[ref64] Kumar P. S.; Hogendoorn J. A.; Feron P. H. M.; Versteeg G. F. Approximate solution to predict the enhancement factor for the reactive absorption of a gas in a liquid flowing through a microporous membrane hollow fiber. J. Membr. Sci. 2003, 213, 231–245. 10.1016/S0376-7388(02)00531-8.

[ref65] Gaspar J.; Fosbøl P. L. A general enhancement factor model for absorption and desorption systems: A CO_2_ capture case-study. Chem. Eng. Sci. 2015, 138, 203–215. 10.1016/j.ces.2015.08.023.

[ref66] Zarca G.; Ortiz I.; Urtiaga A. Kinetics of the carbon monoxide reactive uptake by an imidazolium chlorocuprate(I) ionic liquid. Chem. Eng. J. 2014, 252, 298–304. 10.1016/j.cej.2014.05.011.

[ref67] Siemens gPROMS 7.1.1 https://www.siemens.com/global/en/products/automation/industry-software/gproms-digital-process-design-and-operations.html (Accessed April 2024).

[ref68] McLeod A.; Jefferson B.; McAdam E. Quantifying the loss of methane through secondary gas mass transport (or ‘slip’) from a micro-porous membrane contactor applied to biogas upgrading. Water Res. 2013, 47, 3688–3695. 10.1016/j.watres.2013.04.032. 23726705

[ref69] Saadat M. M.; Norouzbahari S.; Esmaeili M. CO_2_/N_2_ separation by glycerol aqueous solution in a hollow fiber membrane contactor module: CFD simulation and experimental validation. Fuel 2022, 323, 124370 10.1016/j.fuel.2022.124370.

[ref70] Nieminen H.; Järvinen L.; Ruuskanen V.; Laari A.; Koiranen T.; Ahola J. Mass transfer characteristics of a continuously operated hollow-fiber membrane contactor and stripper unit for CO_2_ capture. Int. J. Greenhouse Gas Control 2020, 98, 103063 10.1016/j.ijggc.2020.103063.

