# Supplementary material for: Modeling, Simulation, and Membrane Wetting Estimation in Gas–Liquid Contacting Processes Including Shell-Side Reaction: Biogas Upgrading Using DEA Solution
Source: Ind Eng Chem Res. 2024 May 9;63(20):9185–202. doi: 10.1021/acs.iecr.4c00525 (PMC11190974; doi:10.1021/acs.iecr.4c00525)
Supplement: Supplementary file 1 — ie4c00525_si_001.pdf [file ie4c00525_si_001.pdf]

Supporting Information:
Modelling, simulation and membrane wetting estimation in gas-liquid contacting processes including shell-side reaction: biogas upgrading using DEA solution

Grigorios Pantoleontos*, Dimitrios Koutsonikolas*, Akrivi G. Asimakopoulou, Souzana Lorentzou, George Karagiannakis

Advanced Renewable Technologies & Environmental Materials in Integrated Systems (ARTEMIS) Laboratory, Chemical Process & Energy Resources Institute, Centre for Research & Technology Hellas (CPERI/CERTH), 6th km Charilaou-Thermi, 57001, P.O. Box 361, Thermi, Thessaloniki, Greece

The file includes:

Appendix A – Additional equations for the membrane-based gas absorption system

Appendix B – Physico-chemical properties

Appendix C – Additional literature review, figures and comments

Tables S1, S2, S3

Figures S1, S2

References

* Corresponding authors. Tel.:+30-2310-498414 *Email addresses:* pantole@certh.gr; dkoutson@certh.gr

Appendix A – Additional equations for the membrane-based gas absorption system

For the sake of comparison as part of the membrane modelling canon the following dimensionless quantities are introduced in the current study analysis:

$$\hat{r} = r/R_f,\ \hat{z} = \pi \cdot N_f \cdot z \cdot D/(4Q_{g,in}) = {}^{1}/_{Gz},\ Sh_W = 2K_{ext}R_f/D,\ m^* = mQ_{g,in}/Q_l \tag{S.1}$$

$C_{i,mc}(z)$ is the lumen mixed-cup concentration for every i component defined as:

$$C_{i,mc}(z) = \frac{\int_0^{R_f} 2\pi r\, u_z \cdot C_i(z,r)\partial r}{\int_0^{R_f} 2\pi r\, u_z \partial r} = \frac{4}{R_f^2}\int_0^{R_f} r\left(1-\left(\frac{r}{R_f}\right)^2\right)\cdot C_i(z,r)\partial r \tag{S.2}$$

It has to be noted that the $\hat{z}$ and the m^* parameters are reference values applying the feed (constant) gas mixture flowrate, $Q_{g,in}$, unlike the lumen-side gas mixture average velocity, u, which is defined taking into account the variation of the gas flowrate in the fibers due to pressure drop and mainly the loss of CO_2 through the membrane pores:

$$Q_g(z) = Q_{g,in} - (Q_{g,in} - Q_{g,out})\frac{C_{CO_2,mc}(0) - C_{CO_2,mc}(z)}{C_{CO_2,mc}(0) - C_{CO_2,mc}(L_{eff})}$$
$$Q_{g,avg} = \frac{1}{L_{eff}}\int_0^{L_{eff}} Q_g(z)\,\partial z \tag{S.3}$$

where $Q_{g,in}$, $Q_{g,out}$ are the gas mixture flowrates as measured by the gas flowmeter at the inlet and outlet of the membrane module, respectively, and $Q_{g,avg}$ is the averaged gas flowrate.

The lumen-side gas mixture average velocity is then found to be:

$$u = \frac{Q_{g,avg}}{N_f \pi R_f^2} \tag{S.4}$$

where N_f is the number of fibers. (Note that for ease of calculations the averaged gas flowrate can be taken equal to the mean value of the inlet and outlet flowrates without much difference).

Regarding flow through a bundle of capillary tubes the hydraulic diameter (d_h) concept stems from the Carman-Kozeny theory and it is related to the permeability, K:

$$K = \frac{\varepsilon d_h^2}{16k_k} = \frac{\varepsilon^3}{k_k(1-\varepsilon)^2 A_0^2} \tag{S.5}$$

where k_k is the Kozeny constant, ε is the void fraction of the bed (module) so that it holds $\varepsilon = 1 - \varphi$ (see the main article, Table 1 for the packing fraction, φ), and A_0 is a parameter referring to the geometry of the tubes or particles, with units 1/m [S1].

Following Eq. (S.5) the hydraulic diameter is given by:

$$d_h = \frac{4\varepsilon}{(1-\varepsilon)A_0} \tag{S.6}$$

In Eq. (S.6) the volumetric area adjusted to the solid structure, A_0, is introduced such that $A_0=4/d_{circ}$ for the case of circular cylinder particles of diameter d_{circ}, and $A_0=6/d_p$ for the case of spherical particles of diameter

d_p [S1]. The interstitial (i.e. within the voids) velocity, u_{int}, is related to the superficial (i.e. for a chamber without tubes or particles) velocity, u_{sup}, as:

$$u_{sup} = \varepsilon u_{int} \tag{S.7}$$

For example, given the shell-side volumetric flowrate (i.e. the one measured by the rotameter) the superficial velocity *may* be given by dividing with the whole cross-section of the module (i.e. the one given by the manufacturers – see the main article, Table 1). What is needed is the *interstitial velocity* in the expressions for the dimensionless numbers, e.g. Reynolds number [S2]. In the case of the current study (i.e. circular cylindrical hollow fibers and parallel flow (without inner mixing space as in the cross-flow modules) it holds that:

$$\varepsilon = \frac{V_p}{V_b} = \frac{\pi \frac{d_m^2}{4} L_{eff} - \pi \frac{d_{f,o}^2}{4} N_f L_{eff}}{\pi \frac{d_m^2}{4} L_{eff}} = \frac{d_m^2 - N_f d_{f,o}^2}{d_m^2} \tag{S.8}$$

$$d_h = \frac{d_m^2 - N_f d_{f,o}^2}{N_f d_{f,o}} \tag{S.9}$$

$$a_v = \frac{A_{contact\,surface}}{V_p} = \frac{\pi N_f d_{f,i} L_{eff}}{\pi \frac{d_m^2}{4} L_{eff} - \pi \frac{d_{f,o}^2}{4} N_f L_{eff}} = \frac{4 N_f d_{f,i}}{d_m^2 - N_f d_{f,o}^2} \tag{S.10}$$

where d_m is the diameter of the module, $d_{f,o}$, $d_{f,i}$ are the outer and inner diameters of one fiber, respectively, V_p is the voids volume (shell-side volume accounting for the presence of the bunch of fibers), V_b the inner volume of the module as if it were without fibers, and a_v (units: 1/m) is the ratio of the effective mass transfer area (based on the inner fiber diameter), $A_{contact\,surface}$, to the shell-side voids volume, V_p.
The 1-D postulation in the shell side may resemble the mass transfer behavior in the parallel-flow module used in this study in the absence of significant radial gradients. Computationally, the shell-side mass transfer behavior can be obtained by an 1-D model if all source terms transverse to the parallel flow (i.e. from the fiber zone to the shell side) are included (see e.g. Koutsonikolas et al. [S3] for a similar treatment). For a 2-D shell-side formulation analytical solutions for the velocity profile for parallel-flow modules using Happel's model and Dirichlet BC at the outer diameter of the fiber can be found in [S4]. Happel's free surface model [S5] presupposes that the arrangement of the bundle of cylinders (here: fibers) in a volume (e.g. triangular, square arrays) and the radius of the free-surface postulation are known – see also [S6, S7, S8, S9, S10, S11]. Otherwise, the 1-D model contains all the relevant transfer phenomena that would have been included in a model of higher geometric complexity without further assumptions regarding the shell-side configuration.
The removal efficiency of the absorption process is given by the difference of the gas flowrates – y referring to CO_2:

$$CO_2\ Removal = \frac{y_{in} \cdot Q_{g,in} - y_{out} \cdot Q_{g,out}}{y_{in} \cdot Q_{g,in}} \tag{S.11}$$

Appendix B – Physico-chemical properties

Viscosities and binary diffusivities in the lumen side, $D_{m,j}$, are calculated according to the methodology presented in [S12]. In order to calculate the effective diffusion coefficients in the membrane mesoporous zone of the gas-liquid contact membrane process – in the gas separation process only macroscopic permeance values are needed – it is crucial to calculate Knudsen diffusion coefficients following the resistance-in-series formula [S13]:

$$\frac{1}{D_{eff,j}} = \frac{\tau}{\varepsilon_f}\left(\frac{1}{D_{m,j}} + \frac{1}{D_{k,j}}\right) \tag{S.12}$$

where τ is the tortuosity and ε_f is the porosity of the porous network (provided by the membrane modules supplier).

The Henry's constants for the system CO_2 – aqueous solutions of amines which are inserted to Eq. (6) of the main article are calculated using the N_2O-CO_2 analogy [S14, S15, S16]:

$$H_{CO_2,M} = H_{N_2O,M}\frac{H_{CO_2,W}}{H_{N_2O,W}} \tag{S.13}$$

where $H_{N_2O,W}$ is the Henry's constant of N_2O in water and $H_{CO_2,W}$ is the Henry's constant of CO_2 in water, $H_{N_2O,M}$ is the Henry's constant of N_2O in the aqueous amine solution and $H_{CO_2,M}$ is the Henry's constant of CO_2 in the aqueous amine solution.

The Henry's constant of N_2O and CO_2 in water as a function of temperature is given by [S16] – see Table S.1:

$H_{i,w} = exp(a_i + b_i/T + c_i lnT + d_i T)$ for i=N_2O, CO_2, $H_{i,w}$ in Pa m^3/mol, T in K (S.14)

The Henry's constant of N_2O in pure diethanolamine (DEA) as a function of temperature is given by [S16]:

$H_{N_2O,DEA} = a_{N_2O,DEA} + b_{N_2O,DEA}/T$, H in Pa m^3/mol, T in K (S.15)

The semi-empirical expression for the Henry's constant of N_2O in aqueous DEA solutions is given by – see Table S.1:

$$H_{N_2O,12} = \sum_{i=1}^{2} x_i H_{N_2O,i} + A_{12}(x_1x_2)^2\left(1 - \frac{T}{B_{12}}\right) exp(-C_{12}x_2) \tag{S.16}$$

where 1=water, 2 is DEA, $H_{N_2O,12}$ in Pa m^3/mol, T in K

Table S.1: Parameters for the Henry's constant of N_2O in water and pure amines, for CO_2 in water, and the binary mixture [S16].

Henry's constant [Pa·m³/mol]	A	b	c	d
$H_{N_2O,W}$	158.245	–9048.596	-20.860	–0.00252
$H_{CO_2,W}$	145.369	–8172.355	–19.303	0
$H_{N_2O,DEA}$	–11958.00	50.478	–	–
H_2O (1) + DEA (2)	A_{12}	B_{12}	C_{12}	
	4899600.015	362.079	15.809	

In this study in the absence of relevant literature data the Henry's constant for the system CH_4-DEA is taken as that of the corresponding constant of CH_4 into water, which in any case overestimates the solubility of methane when compared to the presence of an electrolyte or an alkaline solution [S17]. At 25 ^{0}C the Henry's constant for the system CH_4-DEA (~CH_4-H_2O) is taken equal to 0.658 Atm·m³/mol [S18], a value almost similar to those presented by Sander in the author's literature review [S19].

Generally, the temperature dependence of the diffusion coefficients in water can be well approximated by:

$$D_{i,H_2O} = A_i \cdot exp\left(-\frac{E_i}{RT}\right) \tag{S.17}$$

where E_i is the activation energy for diffusion in water. Table S.2 summarizes the preexponential and the activation energy values for the diffusion coefficients of the two gases in water.

Table S.2: Pre-exponential and activation energy values for calculation of gaseous diffusivities in water.

i	E_i (J/mol)	A_i (10^{-6} m²/s)
CO_2 [20]	17618.35	2.350
CH_4 [21]	18364.00	3.047

The diffusivity of CH_4 in aqueous solutions of DEA can be assumed to be equal to the diffusivity in water, while the diffusivity of CO_2 in aqueous solutions of DEA is derived by [S22]:

$$D_{CO_2,DEA} = (1 - 1.1352 \cdot 10^{-7} C_{DEA}) \cdot D_{CO_2,H_2O} \,:\, C_{DEA} \left[\frac{mol}{m^3}\right] \tag{S.18}$$

Diffusivity of DEA in aqueous solutions of DEA is calculated by the following expression [S23]:

$$ln\left(D_{DEA,DEA}\right) = -13.268 - 2287.7/T - 19.699 \cdot 10^{-5} \cdot C_{DEA} \,:\, D_{DEA,DEA} \left[\frac{m^2}{s}\right]$$
$$\text{for } 9 \le C_{DEA} \le 4013 \frac{mol}{\cdot m^3} \text{ and } 298 \le T \le 348\,K \tag{S.19}$$

Snijder et al. provide distinct data for the kinematic viscosity of aqueous solution of DEA at various DEA concentrations and temperatures as seen in Table S.3 [S23]. Regression of these values with multiple variables (T, C_{DEA}) yields the following expression:

$$ln\left(\eta_{DEA,H_2O}\right) = -21.21 + 2179.1142/T + 3.762 \cdot 10^{-4} \cdot C_{DEA} \,:\, \eta_{DEA,H_2O} \left[\frac{m^2}{s}\right] \tag{S.20}$$

for $9 \leq C_{DEA} \leq 4013 \frac{mol}{\cdot m^3}$ and $298 \leq \mathrm{T} \leq 348\ K$

Table S.3: Kinematic viscosity values of aqueous solution of DEA at various DEA concentrations and temperatures [S23].

T [K]	C_{DEA} [mol/m^3]	ηx10^6 [m^2/s]	T [K]	C_{DEA} [mol/m^3]	ηx10^6 [m^2/s]
298	10	0.8826	318	1991	1.1254
298	1007	1.2678	318.1	4011	2.6458
298	1984	1.8500	333	12	0.4780
298	4010	5.0765	333.1	1010	0.6144
308	10	0.7444	333	1995	0.8461
308	1008	0.9939	333	4012	1.8502
308	1989	1.4741	348	9	0.3796
308	4011	3.5941	348.2	1012	0.4784
318	11	0.6059	348	2000	0.6283
317.9	1009	0.8285	348	4013	1.3852

Appendix C – Additional literature review, figures and comments

The gas-liquid contact membrane process considers a mixture (gas or liquid) flowing in the lumen (fiber side), which is confined by the membrane not allowing direct contact of the mixture (liquid or gas) flowing in the outer part of the membrane, in the shell side. The degree of the separation of the mixture into its constituents for this kind of process depends on whether there are reactive conditions between the gas species and the solvent, and not on the membrane which does not exert any particular selectivity to one species over the other [S24, S25].

In the past, for the absorption of CO_2 and especially biogas upgrading various membrane materials have been used in gas-liquid contact membrane processes, such as polypropylene (PP) [S7, S26, S27, S28, S30, S31, S32, S33, S34, S35, S36], polytetrafluoroethylene (PTFE) [S9, S25, S37, S38], polyvinylidenefluoride (PVDF) [S7, S39], polydimethylsiloxane [S40], or mixed-matrix membranes [S41], using H_2O [S25, S26, S32, S40 – see also reviews [S25, S42] and references mentioned therein], NaOH/KOH [S26, S33], NH_3 [S38], ionic liquids [S39] and others [S9, S31, S34, S35, S36, S37] (see also a review by Pantoleontos et al. [S29] of membrane materials and solvents for CO_2 capture). Amine solvents such as the primary amine monoethanolamine (MEA), the secondary amine DEA and the tertiary amine methyldiethanolamine (MDEA), which are preferred in conventional packed towers for CO_2 capture [S43] (see also the evaluation of over 130 aqueous amine solvents regarding carbon-capture performance by Bernhardsen & Knuutila [S30]), are extensively reviewed in membrane-based absorption processes; see e.g. MEA or triethanolamine (TEA) using PVDF membranes [S44], MEA using PP [S45, S46], MEA or DEA using PTFE membranes [S47], blended dimethylethanolamine (DMEA)/MEA in PTFE membranes [S48], aqueous solutions of DEA [S27, S30, S7, S41], MDEA, piperazine (PZ) and 2-amino-2-methyl-1-propanol (AMP) [S31], activated MDEA in PP membranes [S49], investigation of tertiary amines (2-(diethylamino)ethanol –

DEEA, MDEA, dimethylethanolamine – DMEA, and 1-dimethylamino-2-propanol – 1DMA2P) [S50], DMEA using PTFE membranes [S51], single and blended aqueous alkanolamine solutions of MEA, DEA, MDEA and AMP [S52], MEA, DEA, MDEA, AMP and mixed amines using PTFE membranes [S53] and other amine-based solvents [S54, S55].

The performance of CO_2 absorption using different polymeric materials can be ranked as PTFE>PVDF>PP, but the cost of PP is substantially lower than PTFE and PVDF [S56], while PTFE hollow fibers are usually commercially available in larger diameters apparently diminishing the overall specific surface area (see the analysis by Li et al. with suggestions to overcome some issues [S25]). In addition, it is important that chemical solvents react primarily with CO_2 in order to minimize the loss of CH_4 in the solvent [S33], but also to study the possibility of regenerating the solvent and recovering CO_2 at the lowest possible cost.

For a very fast reaction in the shell side the absorption can be treated as an instantaneous reaction, whose rate is immaterial [S57, S58]; for example, the reaction between H_2S and aqueous amines can be regarded as reversible and instantaneously fast, so that everywhere in the liquid an H_2S-amine equilibrium exists [S59] (see also discussion in [S57, S60]), while the shell-side resistance should be very small accounting for a large enhancement factor derived from its asymptotic value, E_∞.

Aqueous DEA solutions may display different kinetic behavior and reaction order with respect to DEA if seen under a wide range of DEA concentrations in the feed solution [S61, and references mentioned therein]. Computationally, it is convenient to use the same reaction rate expression in the whole shell-side compartment instead of using branched functions of rate (and enhancement factors) expressions for decreasing DEA concentrations with the progress of reaction.

The reactions taking place between CO_2 and DEA can be reasonably described by the zwitterion ($R_2NH^+COO^-$) reaction mechanism [S58, S62, S63]:

$$CO_2 + R_2NH \rightleftarrows R_2NH^+COO^-$$
$$R_2NH^+COO^- + R_2NH \rightleftarrows R_2NCOO^- + R_2NH_2^+ \tag{1}$$

with $R = CH_2CH_2OH^-$ for DEA.

Laddha and Danckwerts argue that third order kinetics can be maintained for concentrations up to 0.5M [S64]. Note that the reaction rate values by Hikita et al. [S65] are very close to the values that can be obtained by Alvarez-Fuster et al., who derived a pre-exponential constant of k_R = 840 $L^2/mol^2/s$ (independent of temperature) and an overall third-order reaction rate (as Hikita et al.) for the range of DEA concentrations of 0.25-0.82 M [S66], and the values that can be calculated by the general reaction rate expression by Versteeg et al. [S67, S68].

The enhancement factors in the overall analysis serve as the effect of the chemical reaction on the absorption rate and in principle can be derived by solving a set of equations in the concentration boundary layer (here: of the shell side) where the gaseous components are absorbed in a combination of mechanisms of diffusion and reaction before transported to the liquid bulk [S69]. Thus, the calculation of the dissolved gas concentration involves the solution of the coupled problem of the concentration boundary layer model with the macroscopic model for the liquid bulk including consistent boundary conditions at the computational interfaces [S70]. In the corresponding analysis, the role of different mass transfer models (e.g. film (Nernst-Whitman); penetration and surface-renewal (Higbie and Danckwerts) models [S57]) may not be so crucial since the maximum discrepancy noticed is only a few percent [S57] – still, the determination of the computational wetting depends on the definition of the membrane mass transfer model, see Eqs. (9) and (10) of the main manuscript. Analytical expressions for enhancement factors for second-order reversible reactions [S71] or approximations for n^{th}-order reversible reactions [S72] are available in the literature – see also discussion in [S69, S73, S74, S75].

Figure S1 illustrates the piping and instrumentation diagram of the experimental gas-liquid contact membrane process as adapted from [S76]. The unit setup can be operated either with liquid recycle representing a semi-batch operation mode or on a once-through mode representing a continuous operation mode. The liquid solvents preparation takes place in a 6 L Stainless Steel (SS316) feed tank equipped with a pressure gauge and safety valve. Liquid solvent is being fed with a high precision gear pump (Ismatec ISM446B) through a float ball flowmeter (0-0.5 L/min). Through a 3-way valve, the liquid phase either recirculates into the mixing vessel or is directed to the membrane contactor section. Feed gas is being supplied through two different compressed gas cylinders (containing either single gases or gas mixtures) using two independent Mass Flow Controllers (MFCs) (Bronkhorst F-201CV-20K-AAD-22-V, 1L/min, 5 bar (g)/3 bar (g), CO_2 and Bronkhorst F-201CV-20K-AAD-22-V, 1L/min, 5 bar (g)/3 bar (g), N_2) at flow rates up to approximately 2 L/min (std). Through a series of valves, the feed gas can be either sent directly to the residue/analysis equipment for feed flow and composition measurements or to the membrane module and afterwards to the analysis equipment.

Figure S2 depicts the total membrane mass transfer resistance for CO_2 as calculated by Eq. (8) of the main manuscript with wetting values estimation when applying the Hikita-Costello or the Hikita-Yun pair. A slight deviation from a straight line is due to the slightly different temperatures of the performed experiments. It has to be noted that the individual resistances, gas and liquid, do not depend on the extent of the calculated wetting (thus, on the shell-side mass transfer correlation used) since they are defined as if the whole length of the pore had been filled with either gas or liquid, respectively – subsequently, the estimated wetting value from each correlation is applied to Eq. (8) of the main manuscript to derive the effective membrane mass transfer coefficient, $k_{m,eff}$, whose inverse value, R_m, is the total membrane mass transfer resistance (for CO_2 in Table 4 of the main manuscript).

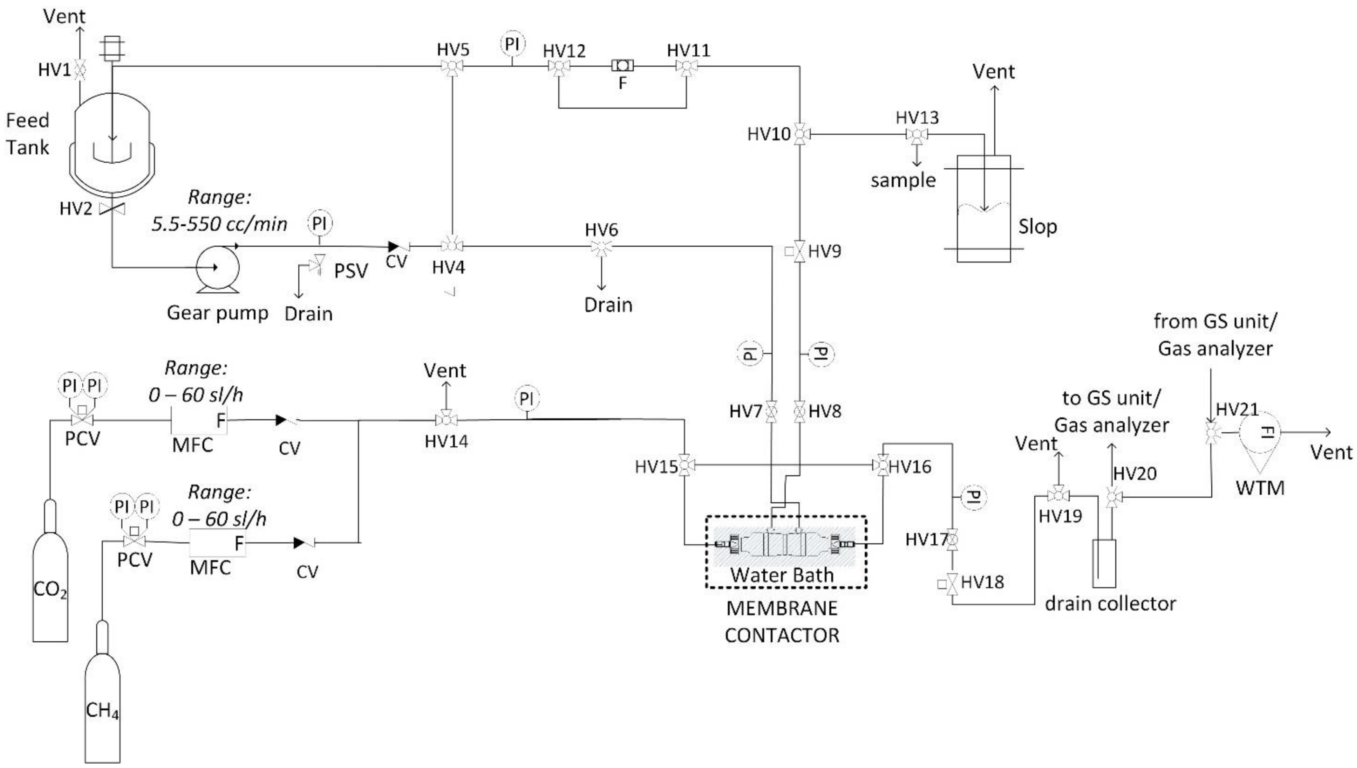

Figure S1. P&ID of the lab-scale experimental unit – adapted from [S76].

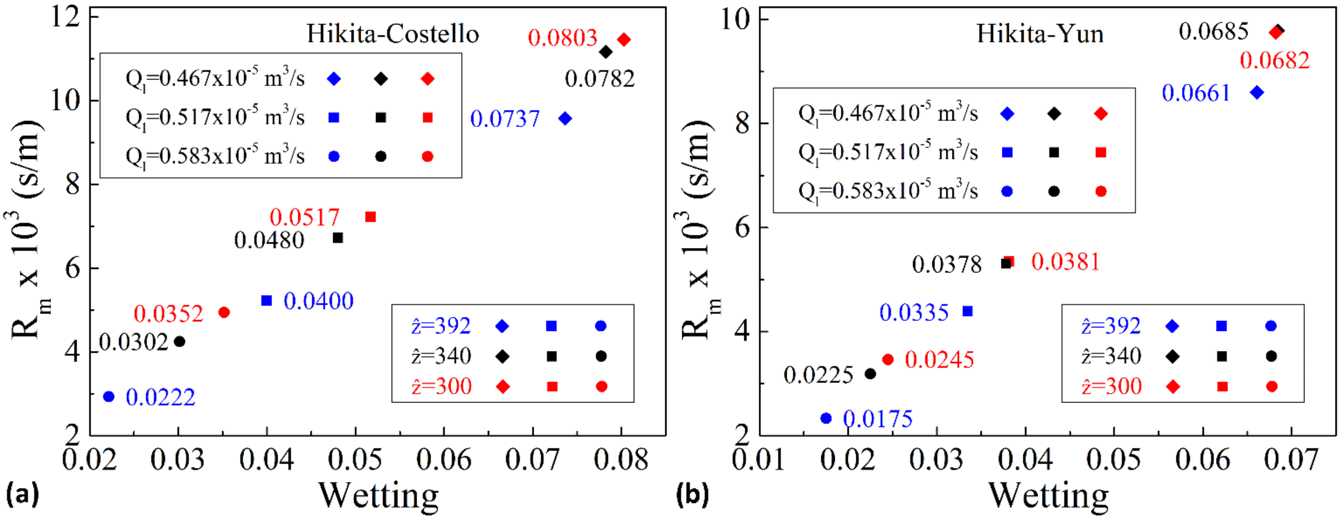

Figure S2. Linear relationship of the total membrane mass transfer resistance of CO_2, R_m, with the wetting for the Hikita-Costello (a) and the Hikita-Yun (b) pairs.

References

S1. Nakayama, A.; Kuwahara, F.; Sano, Y. Concept of equivalent diameter for heat and fluid flow in porous media. *AIChE J.* **2007**, *53*, 732-736.

S2. Cai, J. J.; Hawbolt, K.; Abdi, M. A. Analysis of the effect of module design on gas absorption in cross flow hollow membrane contactors via computational fluid dynamics (CFD) analysis. *J. Membrane Sci.* **2016**, *520*, 415-424.

S3. Koutsonikolas, D. E.; Pantoleontos, G.; Karagiannakis, G.; Konstandopoulos, A. G. Development of H_2 selective silica membranes: Performance evaluation through single gas permeation and gas separation tests. *Sep. Purif. Technol.* **2021**, *264*, 118432

S4. Zheng, J.-M.; Xu, Y.-Y.; Xu, Z.-K. Shell side mass transfer characteristics in a parallel flow hollow fiber membrane module. *Sep. Sci. Tech.* **2003**, *38*, 1247-1267.

S5. Happel, J. Viscous flow relative to arrays of cylinders. *AIChE J.* **1959**, *5*, 174 – 177.

S6. Gong, Y.; Wang, Z.; Wang, S. Experiments and simulation of CO_2 removal by mixed amines in a hollow fiber membrane module. *Chem. Eng. Process.* **2006**, *45*, 652-660.

S7. Zhang, H.-Y.; Wang, R.; Liang, D. T.; Tay, J. H. Theoretical and experimental studies of membrane wetting in the membrane gas-liquid contacting process for CO_2 absorption. *J. Membrane Sci.* **2008**, *308*, 162−170.

S8. El-Naas, M. H.; Al-Marzouqi, M.; Marzouk, S. A.; Abdullatif, N. Evaluation of the removal of CO_2 using membrane contactors: membrane wettability. *J. Membrane Sci.* **2010**, *350*, 410−416.

S9. Cao, F.; Gao, H.; Li, H.; Liang, Z. Experimental and theoretical studies on mass transfer performance for CO_2 absorption into aqueous N,N-dimethylethanolamine solution in the polytetrafluoroethylene hollow-fiber membrane contactor. *Ind. Eng. Chem. Res.* **2018**, *57*, 16862−16874.

S10. Tantikhajorngosol, P.; Laosiripojana, N.; Jiraratananon, R.; Assabumrungrat, S. Physical absorption of CO_2 and H_2S from synthetic biogas at elevated pressures using hollow fiber membrane contactors: The effects of Henry's constants and gas diffusivities. *Int. J. Heat Mass Transfer* **2019**, *128*, 1136-1148.

S11. Sohaib, Q.; Muhammad, A.; Younas, M.; Rezakazemi, M.; Druon-Bocquet, S.; Sanchez-Marcano, J. Rigorous non-isothermal modeling approach for mass and energy transport during CO_2 absorption into aqueous solution of amino acid ionic liquids in hollow fiber membrane contactors. *Sep. Purif. Technol.* **2021**, *254*, 117644.

S12. Pantoleontos, G.; Kikkinides, E. S.; Georgiadis, M. C. A heterogeneous dynamic model for the simulation and optimisation of the steam methane reformer. *Int. J. Hydrogen Energ.* **2012**, *37*, 16346-16358.

S13. Scott, D. S.; Dullien, F. A. L. Diffusion of ideal gases in capillaries and porous solids. *AIChE J.* **1962**, *8*, 113-117.

S14. Clarke, J. K. A. Kinetics of absorption of carbon dioxide in monoethanolamine solutions at short contact times. *Ind. Eng. Chem. Fund.* **1964**, *3*, 239-245.

S15. Sada, E.; Kumazawa, H.; Butt, M. A. Solubilities of gases in aqueous solutions of amine. *J. Chem. Eng. Data* **1977**, *22*, 277-278.

S16. Penttilä, A.; Dell'Era, C.; Uusi-Kyyny, P.; Alopaeus, V. The Henry's law constant of N_2O and CO_2 in aqueous binary and ternary amine solutions (MEA, DEA, DIPA, MDEA, and AMP). *Fluid Phase Equilibr.* **2011**, *311*, 59-66.

S17. Paolini, V.; Torre, M.; Giacopini, W.; Pastori, M.; Segreto, M.; Tomassetti, L.; Carnevale, M.; Gallucci, F.; Petracchini, F.; Guerriero, E. CO_2/CH_4 separation by hot potassium carbonate absorption for biogas upgrading. *Int. J. Greenh. Gas Con.* **2019**, *83*, 186-194.

S18. PubChem, Methane (3.2.21: Other Experimental Properties), National Library of Medicine, National Center for Biotechnology Information.

S19. Sander, R. Compilation of Henry's law constants (version 4.0) for water as solvent. *Atmos. Chem. Phys.* **2015**, *15*, 4399-4981.

S20. Versteeg, G. F.; Van Swaaij, W. P. M. Solubility and diffusivity of acid gases (carbon dioxide, nitrous oxide) in aqueous alkanolamine solutions. *J. Chem. Eng. Data* **1988**, *33*, 29-34.

S21. Jähne, B.; Heinz, G.; Dietrich, W. Measurement of the diffusion coefficients of sparingly soluble gases in water. *J. Geophys. Res.* **1987**, *92*, 767-776.

S22. Karoor, S. Gas separation using microporous hollow fiber membranes, Ph.D. Dissertation, Stevens Institute of Technology, Hoboken, NJ, 1992.

S23. Snijder, E. D.; te Riele, M. J. M.; Versteeg, G. F.; van Swaaij, W. P. M. Diffusion coefficients of several aqueous alkanolamine solutions. *J. Chem. Eng. Data* **1993**, *38*, 475-480.

S24. Xu, Y.; Li, X.; Lin, Y.; Malde, C.; Wang, R. Synthesis of ZIF-8 based composite hollow fiber membrane with a dense skin layer for facilitated biogas upgrading in gas-liquid membrane contactor. *J. Membrane Sci.* **2019**, *585*, 238–252.

S25. Li, M.; Zhu, Z.; Zhou, M.; Jie, X.; Kang, G.; Cao, Y. Removal of CO_2 from biogas by membrane contactor using PTFE hollow fibers with smaller diameter. *J. Membrane Sci.* **2021**, *627*, 119232.

S26. Pantoleontos, G.; Kaldis, S. P.; Koutsonikolas, D.; Grammelis, P.; Sakellaropoulos, G. P. CO_2 absorption in a mini-module membrane contactor. In *Global Warming*. Springer Science+Business Media, LLC, 2010; pp. 307–313.

S27. Koutsonikolas, D.; Pantoleontos, G.; Mavroudi, M.; Kaldis, S.; Pagana, A.; Kikkinides, E. S.; Konstantinidis, D. Pilot tests of CO_2 capture in brick production industry using gas–liquid contact membranes. *Int. J. Energ. Environ. Eng.* **2016**, *7*, 61−68.

S28. Pantoleontos, G.; Theodoridis, T.; Mavroudi, M.; Kikkinides, E. S.; Koutsonikolas, D.; Kaldis, S. P.; Pagana, A. E. Modelling, simulation, and membrane wetting estimation in gas-liquid contacting processes. *Can. J. Chem. Eng.* **2017**, *95*, 1352–1363.

S29. Pantoleontos, G.; Anagnostara, I. M.; Syrigou, M.; Konstandopoulos, A. G. Solutions of the mass continuity equation in hollow fibers for fully developed flow with some notes on the Lévêque correlation. *Carbon Capt. Sci. Technol.* **2022**, *2*, 100027.

S30. Zhang, H.-Y.; Wang, R.; Liang, D. T.; Tay, J. H. Modeling and experimental study of CO_2 absorption in a hollow fiber membrane contactor. *J. Membrane Sci.* **2006**, *279*, 301−310.

S31. Lu, J.-G.; Zheng, Y.-F.; Cheng, M.-D.; Wang, L.-J. Effects of activators on mass-transfer enhancement in a hollow fiber contactor using activated alkanolamine solutions. *J. Membrane Sci.* **2007**, *289*, 138−149.

S32. Mavroudi, M.; Kaldis, S. P.; Sakellaropoulos, G. P. Effect of flow configuration on the performance of gas–liquid membrane contactor. *Procedia Eng.* **2012**, *44*, 1454−1457.

S33. McLeod, A.; Jefferson, B.; McAdam, E. Quantifying the loss of methane through secondary gas mass transport (or 'slip') from a micro-porous membrane contactor applied to biogas upgrading. *Water Res.* **2013**, *47*, 3688−3695.

S34. Yan, S.; He, Q.; Zhao, S.; Wang, Z.; Ai, P. Biogas upgrading by CO_2 removal with a highly selective natural amino acid salt in gas–liquid membrane contactor. *Chem. Eng. Process.* **2014**, *85*, 125−135.

S35. Nieminen, H.; Järvinen, L.; Ruuskanen, V.; Laari, A.; Koiranen, T.; Ahola, J. Mass transfer characteristics of a continuously operated hollow-fiber membrane contactor and stripper unit for CO_2 capture. *Int. J. Greenh. Gas Con.* **2020**, *98*, 103063.

S36. Asimakopoulou, A.; Koutsonikolas, D.; Kastrinaki, G.; Skevis, G. Innovative gas-liquid membrane contactor systems for carbon capture and mineralization in energy intensive industries. *Membranes* **2021**, *11*, 271.

S37. Kumar, P. S.; Hogendoorn, J. A.; Feron, P. H. M.; Versteeg, G. F. New absorption liquids for the removal of CO_2 from dilute gas streams using membrane contactors. *Chem. Eng. Sci.* **2002**, *57*, 1639–1651.

S38. McLeod, A.; Buzatu, P.; Autin, O.; Jefferson, B.; McAdam, E. Controlling shell-side crystal nucleation in a gas–liquid membrane contactor for simultaneous ammonium bicarbonate recovery and biogas upgrading. *J. Membrane Sci.* **2015**, *473*, 146−156.

S39. Gomez-Coma, L.; Garea, A.; Irabien, A. Carbon dioxide capture by [emim][Ac] ionic liquid in a polysulfone hollow fiber contactor. *Int. J. Greenh. Gas Con.* **2016**, *52*, 401−409.

S40. Heile, S.; Rosenberger, S.; Parker, A.; Jefferson, B.; McAdam, E. J. Establishing the suitability of symmetric ultrathin wall polydimethylsiloxane hollow-fibre membrane contactors for enhanced CO_2 separation during biogas upgrading. *J. Membrane Sci.* **2014**, *452*, 37−45.

S41. Gong, H.; Pang, H.; Du, M.; Chen, Z. Fabrication of a superhydrophobic mixed matrix PVDF-SiO_2-HDTMS hollow fiber membrane for membrane contact carbon dioxide absorption. *Cleaner Eng. Tech.* **2021**, *5*, 100278.

S42. Belaissaoui, B.; Claveria-Baro, J.; Lorenzo-Hernando, A.; Zaidiza, D. A.; Chabanon, E.; Castel, C.; Rode, S.; Roizard, D.; Favre, E. Potentialities of a dense skin hollow fiber membrane contactor for biogas purification by pressurized water absorption. *J. Membrane Sci.* **2016**, *513*, 236−249.

S43. Wang, Z.; Fang, M.; Yan, S.; Yu, H.; Wei, C.-C.; Luo, Z. Optimization of blended amines for CO_2 absorption in a hollow-fiber membrane contactor. *Ind. Eng. Chem. Res.* **2013**, *52*, 12170−12182.

S44. Yeon, S.-H.; Lee, K.-S.; Sea, B.; Park, Y.-I.; Lee, K.-H. Application of pilot-scale membrane contactor hybrid system for removal of carbon dioxide from flue gas. *J. Membrane Sci.* **2005**, *257*, 156−160.

S45. Xu, Y.; Malde, C.; Wang, R. Correlating physicochemical properties of commercial membranes with CO_2 absorption performance in gas-liquid membrane contactor. *J. Membrane Sci. Res.* **2006**, *6*, 30−39.

S46. deMontigny, D.; Tontiwachwuthikul, P.; Chakma, A. Using polypropylene and polytetrafluoroethylene membranes in a membrane contactor for CO_2 absorption. *J. Membrane Sci.* **2006**, *277*, 99−107.

S47. Constantinou; A. Barrass, S.; Gavriilidis, A. CO_2 absorption in polytetrafluoroethylene membrane microstructured contactor using aqueous solutions of amines. *Ind. Eng. Chem. Res.* **2014**, *53*, 9236-9242.

S48. Zhang, P.; Xu, R.; Li, H.; Gao, H.; Liang, Z. Mass transfer performance for CO_2 absorption into aqueous blended DMEA/MEA solution with optimized molar ratio in a hollow fiber membrane contactor. *Sep. Purif. Technol.* **2019**, *211*, 628-636.

S49. Akan, A. P.; Chau, J.; Sirkar, K. K. Post-combustion CO_2 capture and recovery by pure activated methyldiethanolamine in crossflow membrane contactors having coated hollow fibers. *Sep. Purif. Technol.* **2020**, *244*, 116427.

S50. Yin, Y.; Cao, Z.; Gao, H.; Sema, T.; Na, Y.; Xiao, M.; Liang, Z.; Tontiwachwuthikul, P. Experimental measurement and modeling prediction of mass transfer in a hollow fiber membrane contactor using tertiary amine solutions for CO_2 absorption. *Ind. Eng. Chem. Res.* **2022**, *61*, 9632−9643.

S51. Cao, F.; Gao, H.; Xiong, Q.; Liang, Z. Experimental studies on mass transfer performance for CO_2 absorption into aqueous N,N-dimethylethanolamine (DMEA) based solutions in a PTFE hollow fiber membrane contactor. *Int. J. Greenh. Gas Con.* **2019**, *82*, 210-217.

S52. Paul, S.; Ghoshal; A. K.; Mandal, B. Removal of CO_2 by single and blended aqueous alkanolamine solvents in hollow-fiber membrane contactor: modeling and simulation. *Ind. Eng. Chem. Res.* **2007**, *46*, 2576-2588.

S53. Iliuta, I.; Bougie, F.; Iliuta, M. C. CO_2 removal by single and mixed amines in a hollow-fiber membrane module–investigation of contactor performance. *AIChE J.* **2015**, *61*, 955-971.

S54. Chen, G.; Chen, G.; Cao, F.; Zhang, R.; Gao, H.; Liang, Z. Mass transfer performance and correlation for CO_2 absorption into aqueous 3-diethylaminopropylamine solution in a hollow fiber membrane contactor. *Chem. Eng. Process.* **2020**, *152*, 107932.

S55. Gao, H.; Liu, S.; Gao, G.; Luo, X.; Liang, Z. Hybrid behavior and mass transfer performance for absorption of CO_2 into aqueous DEEA/PZ solutions in a hollow fiber membrane contactor. *Sep. Purif. Technol.* **2018**, *201*, 291-300.

S56. Khaisri, S.; deMontigny, D.; Tontiwachwuthikul, P.; Jiraratananon, R. Comparing membrane resistance and absorption performance of three different membranes in a gas absorption membrane contactor. *Sep. Purif. Technol.* **2009**, *65*, 290-297.

S57. Danckwerts, P. V. *Gas-liquid reactions*, McGraw-Hill, 1970.

S58. Versteeg, G. F., van Diijk, L. A. J., van Swaaij, W. P. M. On the kinetics between CO_2 and alkanolamines both in aqueous and non-aqueous solutions. An overview. *Chem. Eng. Commun.* **1996**, *144*, 113−158.

S59. Mandal, B. P.; Bandyopadhyay, S. S. Simultaneous absorption of carbon dioxide and hydrogen sulfide into aqueous blends of 2-amino-2-methyl-1-propanol and diethanolamine. *Chem. Eng. Sci.* **2005**, *60*, 6438−6451.

S60. Qin, Y.; Cabral, J. M. S.; Wang, S. Hollow-fiber gas-membrane process for removal of NH_3 from solution of NH_3 and CO_2. *AIChE J.* **1996**, *42*, 1945−1956.

S61. Mahajani, V. V.; Joshi, J. B. Kinetics of reactions between carbon dioxide and alkanolamines. *Gas Sep. Purif.* **1988**, *2*, 50−64.

S62. Danckwerts, P. V. The reaction of CO_2 with ethanolamines. *Chem. Eng. Sci.* **1979**, *34*, 443−446.

S63. Littel, R. J.; Versteeg, G. F.; van Swaaij, W. P. M. Kinetics of CO_2 with primary and secondary amines in aqueous solutions—I. Zwitterion deprotonation kinetics for DEA and DIPA in aqueous blends of alkanolamines. *Chem. Eng. Sci.* **1992**, *47*, 2027−2035.

S64. Laddha, S. S.; Danckwerts, P. V. Reaction of CO_2 with ethanolamines: kinetics from gas-absorption. *Chem. Eng. Sci.* **1981**, *36*, 479−482.

S65. Hikita, H.; Asai, S.; Ishikawa, H.; Honda, M. The kinetics of reactions of carbon dioxide with monoethanolamine, diethanolamine and triethanolamine by a rapid mixing method. *Chem. Eng. J.* **1977**, *13*, 7−12.

S66. Alvarez-Fuster, C.; Midoux, N.; Laurent, A.; Charpentier, J. C. Chemical kinetics of the reaction of carbon dioxide with amines in pseudo m-nth order conditions in aqueous and organic solutions. *Chem. Eng. Sci.* **1980**, *35*, 1717−1723.

S67. Versteeg, G. F.; van Swaaij, W. P. M. On the kinetics between CO_2 and alkanolamines both in aqueous and non-aqueous solutions—I. Primary and secondary amines. *Chem. Eng. Sci.* **1988**, *43*, 573−585.

S68. Versteeg, G. F.; Oyevaar, M. H. The reaction between CO_2 and diethanolamine at 298 K. *Chem. Eng. Sci.* **1989**, *44*, 1264−1268.

S69. Mirzaei, F.; Ghaemi, A. An experimental correlation for mass transfer flux of CO_2 reactive absorption into aqueous MEA-PZ blended solution. *Asia Pac. J. Chem. Eng.* **2018**, *13*, e2250.

S70. Ramachandran, P. A. *Mass transfer processes. Modelling, computations, and design*; Prentice Hall, 2018.

S71. DeCoursey, W. J. Enhancement factors for gas absorption with reversible reaction. *Chem. Eng. Sci.* **1982**, *37*, 1483−1489.

S72. Hogendoorn, J. A.; Vas Bhat, R. D.; Kuipers, J. A. M.; van Swaaij, W. P. M.; Versteeg, G. F. Approximation for the enhancement factor applicable to reversible reactions of finite rate in chemically loaded solutions. *Chem. Eng. Sci.* **1997**, *52*, 4547−4559.

S73. Kumar, P. S.; Hogendoorn, J. A.; Feron, P. H. M.; Versteeg, G. F. Approximate solution to predict the enhancement factor for the reactive absorption of a gas in a liquid flowing through a microporous membrane hollow fiber. *J. Membrane Sci.* **2003**, *213*, 231−245.

S74. Gaspar, J.; Fosbøl, P. L. A general enhancement factor model for absorption and desorption systems: A CO_2 capture case-study. *Chem. Eng. Sci.* **2015**, *138*, 203−215.

S75. Putta, K. R.; Tobiesen, F. A.; Svendsen, H. F.; Knuutila, H. K. Applicability of enhancement factor models for CO_2 absorption into aqueous MEA solutions. *Appl. Energ.* **2017**, *206*, 765−783.

S76. Asimakopoulou, A.; Koutsonikolas, D.; Kastrinaki, G.; Skevis, G. Innovative gas-liquid membrane contactor systems for carbon capture and mineralization in energy intensive industries. *Membranes* **2021**, *11*, 271.
